# Exploring the scope of DBU-promoted amidations of 7-methoxycarbonylpterin

**DOI:** 10.3762/bjoc.16.46

**Published:** 2020-03-26

**Authors:** Anna R Bockman, Jeffrey M Pruet

**Affiliations:** 1 Department of Chemistry, Valparaiso University, 1710 Chapel Dr, Valparaiso, IN 46383, USA

**Keywords:** amidation, DBU, folate, pterin

## Abstract

The synthetic utility of pterins is often hampered by the notorious insolubility of this heterocycle, slowing the development of medicinally relevant pteridine derivatives. Reactions which expedite the development of new pterins are thus of great importance. Through a dual role of diazabicycloundecene (DBU), 7-carboxymethylpterin is converted to the soluble DBU salt, with additional DBU promoting an ester-to-amide transformation. We have explored this reaction to assess its scope and identify structural features in the amines which significantly affect success, monitored the reaction kinetics using a pseudo-first order kinetics model, and further adapted the reaction conditions to allow for product formation in as little as 5 min, with yields often >80%.

## Introduction

Pteridines are a class of fused bicyclic heterocycles with significant biological relevance. The 2-amino-4-pteridinone core, simply referred to as “pterin”, is present in various biological cofactors like folates, biopterin, and molybdopterin [1]. Owing to this biological relevance, pterins are an attractive building block in the development of various pharmaceuticals, with methotrexate being the most well-known [2]. In addition to pteridine derivatives being used as inhibitors of dihydrofolate reductase and dihydropteroate synthase, pterins have been a useful scaffold in the development of inhibitors of ricin [3–4], methionine synthase [5], nitric oxide synthase [6], shigella [7], and aldose reductase [8], as well as for treatment of leishmaniosis [9]. In addition to their therapeutic potential, pterin derivatives have been an attractive scaffold in the field of supramolecular chemistry, due to their well-defined donor/acceptor hydrogen-bonding arrays [10–11].

One limitation often encountered with the synthesis of structurally diverse pterins is the notorious insolubility in most solvents. This can be dealt with by preemptive modifications to the pteridine core which disrupt the hydrogen-bond assembly, such as benzylation of the lactam oxygen or conversion of the exocyclic amine to the pivalic amide [12–13]. The installation and subsequent removal of these groups naturally affects the overall yield and time necessary to generate new pterins. As such, any reaction which expedites the synthesis of pterin derivatives, especially those which bypass the insolubility, is of great importance to both medicinal and supramolecular chemists.

**Figure 1:**
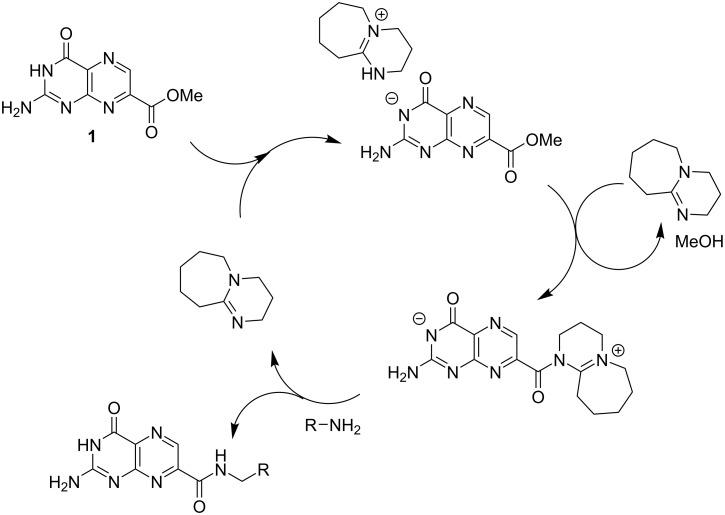
The dual role of DBU in the amidation of 7-CMP.

We first reported the application of DBU as a crucial additive in the synthesis of pterins used for ricin toxin A (RTA) inhibitors [14]. By deprotonation of the lactam NH, and conversion to the DBU salt, the pterin easily dissolves in methanol at high concentrations, unprecedented for unfunctionalized pterins. This greatly accelerated the development of a library of bioactive pterins, as it bypassed the problematic insolubility and assisted in derivatization [14–16]. In addition to this improvement in solubility, we can further take advantage of a mechanistic role of DBU, as it catalyzes the ester-to-amide transformation, allowing for 7-methoxycarbonylpterin (7-CMP, **1**) to smoothly generate new pterin amides (Figure 1) [14,17]. The same process used in generating RTA inhibitors was later used as a key step in the development of new aldolase reductase inhibitors [8]. The benefit of DBU as an additive in these previous reports was largely empirical, and reaction times were still in the order of several hours.

Viewing this reaction as an essential tool for pteridine chemists seeking to rapidly generate diverse pterin structures, we sought to explore the scope of amines which can be easily appended to the pterin core. Trends in reactivity were further probed by monitoring the reaction by employing a pseudo-first order kinetics model. Furthermore, we adapted the reaction to allow for reaction times as little as 5 minutes, with yields reaching up to 99%.

## Results and Discussion

**Scheme 1:**
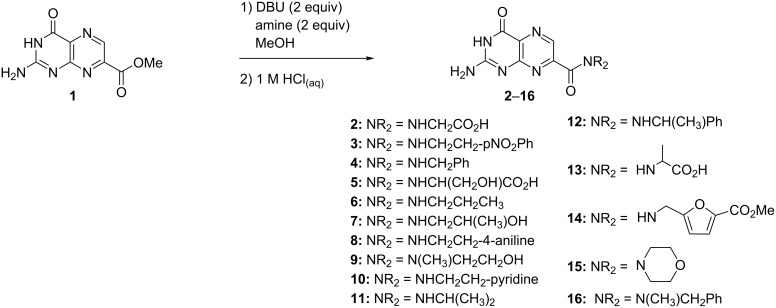
DBU-promoted amidation of 7-CMP (**1**).

The general procedure for the DBU-promoted amidation involved the initial suspension of 7-CMP (**1**) in a small quantity of anhydrous methanol, followed by the addition of 2 equiv DBU resulting in the full dissolution of the pterin ester, whereupon 2 equiv of the amine of interest were added and the reaction was stirred until complete (Scheme 1). The isolation of the amide products **2**–**16** was achieved by careful acidification with dilute aqueous HCl. By reversing the DBU-salt formation, the pterin turns again insoluble and cleanly precipitates, allowing for an isolation by simple filtration.

Initially, the reaction was allowed to proceed at room temperature overnight to ensure adequate times for all amines screened to react. This preliminary screen of amines gave acceptable yields for a range of substituted amines, with primary amines often giving the best results, as expected. To streamline the amine screening and allow for better comparisons of reactivity, a variety of times and temperatures were screened with benzylamine chosen as a representative example (i.e., formation of product **4**) in the method optimization (Table 1). It quickly became apparent that simple amines easily gave rise to the amide under mild reaction conditions (5 min at 60 °C). Using this modified method allowed for all screened amines to be adequately reassessed on their ease of amidation (Table 2).

**Table 1:** Optimizing the conditions with benzylamine.

Temp (°C)	Time (min)	Yield (%)

110	30	78
110	20	90
80	30	89
80	10	90
60	10	91
60	5	91
60	1	44
40	5	46

**Table 2:** Summary of amines screened for amidation of 7-CMP.

Product	Amine tested	Conditions^a^	Yield (%)

**2**	glycine	60 °C, 5 min	99
**3**	4-nitrophenethylamine	60 °C, 5 min	96
**4**	benzylamine	60 °C, 5 min	91
**5**	serine	60 °C, 5 min	88
**6**	1-propylamine	60 °C, 5 min	87
**7**	1-amino-2-propanol	60 °C, 5 min	85
**8**	4-(2-aminoethyl)aniline	60 °C, 5 min	76
**9**	2*-*(methylamino)ethanol	60 °C, 5 min	71
**10**	2-(2-aminoethyl)pyridine	60 °C, 5 min	66
**11**	isopropylamine	80 °C, 10 min	66
**12**	α-methylbenzylamine	80 °C, 10 min	60
**13**	alanine	80 °C, 10 min	49
**14**	methyl 2-(aminomethyl)-5-furanoate	60 °C, 5 min	47^b^
**15**	morpholine	80 °C, 20 min	44
**16**	*N*-methylbenzylamine	130 °C, 3 h	63^c^

^a^Typical conditions: 2 equiv amine, 2 equiv DBU, sealed reaction vessel. ^b^The low yield for this amine was due to a side reaction. ^c^No reaction under typical conditions. 10 equiv amine were used.

**Figure 2:**
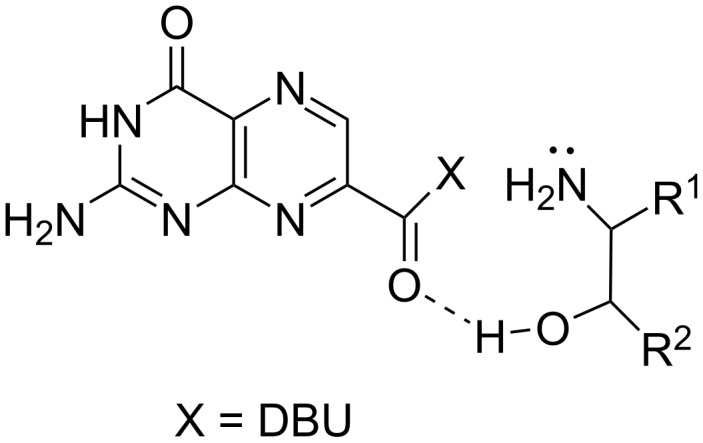
Role of a β-hydroxy group in aiding the amidation reaction.

It was found that the reaction was most affected by an α-substitution on the amine (Table 2; products **11**–**13**). This is in agreement with known Taft parameters which show steric effects are more pronounced for an isopropyl group compared to a benzyl substituent [18]. The deleterious effect of a substituent α to the amine was most pronounced in the case of alanine (product **13**), as compared to glycine (product **2**). However, this steric constraint could be largely overcome by the presence of a β-hydroxy group, as seen with serine (product **5**). For β-hydroxyamines it is expected the hydroxy group expedites the amidation via hydrogen bonding to the 7-CMP carbonyl group, to assist in bringing the amine nucleophile into place (Figure 2). Quite unsurprisingly, secondary amines were less reactive, with *N*-methylbenzylamine (for **16**) being entirely unreactive under the typical reaction conditions. In this case the reaction required 3 h at 130 °C before product formation was observed. Interestingly, much like the improved results for serine, a β-hydroxy group dramatically overcame this issue for secondary amines, as 2-(methylamino)ethanol smoothly reacted affording product **9** in good yield under reaction conditions identical to those for the unhindered primary amines (5 min, 60 °C). Consistent with previous NMR studies of secondary amides, each of these were observed as a resolved mixture of the *s-cis* and *s-trans* rotamers [19]. Product **16** obtained from *N*-methylbenzylamine was found to be a near 1-to-1 mixture of rotamers, while product **9** from 2-(methylamino)ethanol appeared as a 2-to-1 mixture favoring the *s-cis* form.

Another noteworthy entry from Table 2 was methyl 2-aminomethyl-5-furanoate (for **14**). As the DBU amidation relies on an activation of the methyl ester of 7-CMP (**1**), the use of an amine with an additional ester functionality can therefore pose a limitation in what this reaction may tolerate. Yields were diminished for this reaction, owing to a side reaction whereby the amine effectively polymerizes. However, the desired product was still accessible within a short reaction time, and easily purified [20]. This highlighted a limitation of the reaction, but nevertheless showed the reaction may be used in tethering additional reactive functionality to the pterin for future diversification.

**Figure 3:**
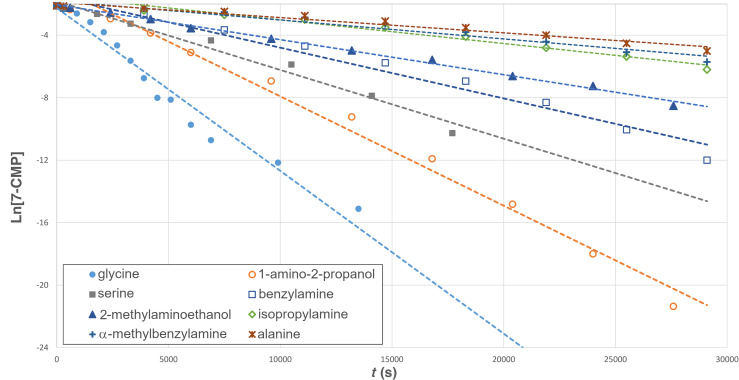
Pseudo-first order kinetics for representative amines.

In an effort to better understand the relative reactivity of the various amines, a pseudo-first order kinetics of the amidation reaction was probed. The reaction was followed by ^1^H NMR spectroscopy at room temperature, following the disappearance of 7-CMP and appearance of the amide product (Figure 3). By monitoring the reaction within the NMR tube with a large excess of the amine, the relative rate constants were determined for a representative set of amines (Table 3). These results further highlight the dramatic rate enhancement brought on by substituent effects. Glycine reacted most swiftly, as the carboxylate anion likely ion pairs with the cationic DBU-activated acyl intermediate (see Figure 1). However, the α-substitution in alanine clearly abolishes any rate acceleration driven by ionic interactions, and most α-substituted amines had similar rates. Rate acceleration is recovered in serine, owing to the effect of the β-hydroxy group discussed previously. The 1-amino-2-propanol reacted faster than serine, likely due to a lack of α-substitution. The effect of the β-hydroxy group was again seen in the swift rate of 2-(methylamino)ethanol. This was in stark contrast to the effectively unreactive *N*-methylbenzylamine which required extreme forcing conditions to react.

**Table 3:** Rate constants determined by ^1^H NMR kinetics.^a^

Amine	*k* @ 22 °C (M^−1^ s^−1^)	Amine	*k* @ 22 °C (M^−1^ s^−1^)

glycine	3.4 × 10^−3^	2-(methylamino)ethanol	0.78 × 10^−3^
1-amino-2-propanol	2.3 × 10^−3^	isopropylamine	0.61 × 10^−3^
serine	1.5 × 10^−3^	α-methylbenzylamine	0.57 × 10^−3^
benzylamine	1.1 × 10^−3^	alanine	0.45 × 10^−3^

^a^Values determined by first finding *k*_obs_ from pseudo-first order kinetic plot.

## Conclusion

The DBU-promoted amidation of 7-methoxycarbonylpterin is a useful method for rapidly generating new pterin derivatives, for medicinal or supramolecular purposes. A paramount benefit of this reaction is its ability to bypass the notorious insolubility of pterins in most solvents. We have shown that this reaction also benefits in its ease and often rapid reaction times (typically 5–10 min). While this amidation reaction can be hindered by typical steric effects, we have shown these issues are largely overcome in amines with additional hydrogen-bonding substituents like a β-hydroxy group. As such, the DBU amidation of 7-CMP can be viewed as an essential tool for heterocyclic chemists.

## Supporting Information

File 1 General procedures, characterization data, and copies of NMR spectra.
