# Supplementary material for: Exploring the scope of DBU-promoted amidations of 7-methoxycarbonylpterin
Source: Beilstein J Org Chem. 2020 Mar 26;16:509–14. doi: 10.3762/bjoc.16.46 (PMC7113547; doi:10.3762/bjoc.16.46)
Supplement: File 1 — General procedures, characterization data, and copies of NMR spectra. [file Beilstein_J_Org_Chem-16-509-s001.pdf]

BEILSTEIN JOURNAL OF ORGANIC CHEMISTRY

Supporting Information

for

Exploring the scope of DBU-promoted amidations of 7-methoxycarbonylpterin

Anna R. Bockman and Jeffrey M. Pruet

Beilstein J. Org. Chem. **2020,** *16,* 509–514. doi:10.3762/bjoc.16.46

General procedures, characterization data, and copies of NMR spectra

License and Terms: This is a supporting information file under the terms of the Creative Commons Attribution License (http://creativecommons.org/licenses/by/4.0). Please note that the reuse, redistribution and reproduction in particular requires that the authors and source are credited.
The license is subject to the *Beilstein Journal of Organic Chemistry* terms and conditions: (https://www.beilstein-journals.org/bjoc)

Table of contents

General information S1
Synthesis S1
Pseudo-first order kinetics S5
Copies of ^{1}H and ^{13}C NMR spectra S6
References S21

General information:

All reagents used were of commercial quality and obtained from Aldrich Chemical Co. and used as received. ^{1}H and ^{13}C NMR spectra were recorded in DMSO-d_6 (or methanol-d_4 for NMR kinetics) with a Bruker spectrometer using the solvent as the reference. Chemical shifts are given in parts per million (ppm). IR data were collected with a NEXUS 670 FT-IR. Purity was assessed on a Waters UPLC with a QDa detector. High resolution mass spectrometry was performed with a Varian 9.4T QFT-ESI ICR system. Filtrations were performed by aid of reduced pressure. Amidation reactions were run using an Anton-Paar MonoWave50™ reactor, operated without microwave irradiation and simply worked as a controlled heating block.[1]

Synthesis

7-Methoxycarbonylpterin (1): Compound **1** was prepared by our previously reported acyl-radical insertion reaction.[2]

General method for the DBU-assisted amidation:
Compound **1** (50 mg, 0.23 mmol) was suspended in 1.0 mL of anhydrous MeOH in an oven-dried borosilicate reaction vessel (Anton-Paar), and stirred to create a slurry. To this was added DBU (68 μL, 0.45 mmol, 2 equiv) which resulted in full dissolution of **1**. To this was added the amine (2 equiv). (**Note:** for amines containing an acidic functional group, e.g. Gly/Ser/Ala, 4 equiv DBU were used to ensure basic conditions). The reaction vessel was then fitted with a silicone cap containing a PTFE seal. The tube was placed in a MonoWave50™ reactor and irradiated for the time and temperature indicated. Once the reaction was complete, the mixture was diluted with 1 mL of DI water, and acidified by the dropwise addition of 3 M HCl, resulting in a yellow precipitate. The product was isolated by filtration, rinsed several times with water and MeOH, and dried over P_2O_5 in a vacuum oven.

(2-Amino-4-oxo-3,4-dihydropteridine-7-carbonyl)glycine (2): Compound **2** was synthesized by the general method above, using 34 mg glycine and 4 equiv DBU, resulting in 59 mg (99%) **2**. MP > 300°C. ^{1}H NMR (400 MHz, DMSO-d_6) δ(ppm)= 12.72 (br, 1H),

11.61 (s, 1H), 9.04 (t, *J* = 5.6 Hz, 1H), 8.88 (s, 1H), 7.03 (br, 2H), 3.99 (d, *J* = 6.0 Hz); ^{13}C NMR (100 MHz, DMSO-d_6) δ(ppm)= 171.2, 163.5, 160.8, 156.5, 154.9, 147.6, 136.9, 132.4, 41.5; HRMS-ESI (m/z) $[M–H]^-$ calc. for $(C_9H_7N_6O_4)^-$ 263.0534; found 263.0533. This is consistent with that reported in the literature.[3]

2-Amino-*N*-(4-nitrophenethyl)-4-oxo-3,4-dihydropteridine-7-carboxamide (3): Compound **3** was synthesized by the general method, using 91 mg of 4-nitrophenethylamine hydrochloride and 4 equiv DBU, resulting in 77 mg (96%) of **3**. MP > 300°C. ^1H NMR (400 MHz, DMSO-d_6) δ(ppm)= 11.58 (s, 1H), 8.98 (t, *J* = 6 Hz, 1H), 8.83 (s, 1H), 8.15 (d, *J* = 8.8 Hz, 2H), 7.54 (d, *J* = 8.8 Hz, 2H), 6.98 (br, 2H), 3.62 (q, *J* = 6.8 Hz, 2H), 3.04 (t, *J* = 7 Hz, 2H); ^{13}C NMR (100 MHz, DMSO-d_6) δ(ppm)= 163.3, 160.8, 156.5, 154.8, 148.3, 148.1, 146.6, 137.0, 132.1, 130.5, 123.9, 40.8, 35.1; IR (cm^{-1}): 3414, 3216, 1722, 1637, 1514, 1342; HRMS-ESI (m/z) $[M+H]^+$ calc. for $(C_{15}H_{14}N_7O_4)^+$ 356.1102; found 356.1101

2-Amino-*N*-benzyl-4-oxo-3,4-dihydropteridine-7-carboxamide (4): Compound **4** was synthesized by the general method, using 50 μL of benzylamine and 2 equiv DBU, resulting in 64 mg (91%) of **4**. MP > 300°C. ^1H NMR (400 MHz, DMSO-d_6) δ(ppm)= 11.63 (s, 1H), 9.41 (t, *J* = 6.3 Hz, 1H), 8.88 (s, 1H), 7.35-7.2 (m, 5H), 7.01 (br, 2H), 4.57 (d, *J* = 6 Hz, 2H); ^{13}C NMR (100 MHz, DMSO-d_6) δ(ppm)= 163.4, 160.8, 156.6, 154.8, 148.3, 139.6, 137.2, 132.1, 128.7 (2C), 127.8 (2C), 127.3, 42.9; IR (cm^{-1}): 3434, 3321, 3189, 1713, 1635, 1518, 1222, 772; HRMS-ESI (m/z) $[M+H]^+$ calc. for $(C_{14}H_{13}N_6O_2)^+$ 297.1094; found 297.1095

(2-Amino-4-oxo-3,4-dihydropteridine-7-carbonyl)serine (5): Compound **5** was synthesized by the general method above, using 48 mg serine and 4 equiv DBU, resulting in 58 mg (88%) **5**. MP > 300°C. ^1H NMR (400 MHz, DMSO-d_6) δ(ppm)= 13.02 (br, 1H), 11.65 (s, 1H), 8.89 (s, 1H), 8.68 (d, *J* = 7.9 Hz, 1H), 7.05 (br, 2H), 5.27 (br. s, 1H), 4.50 (dt, *J* = 7.9, 3.6 Hz, 1H), 3.93-3.79 (m, 2H); ^{13}C NMR (100 MHz, DMSO-d_6) δ(ppm)= 171.9, 162.7, 160.8, 156.4, 155.0, 147.2, 136.7, 132.6, 61.5, 55.2; HRMS-ESI (m/z) $[M–H]^-$ calc. for $(C_{10}H_9N_6O_5)^-$ 293.0640; found 293.0638. This is consistent with that reported in the literature.[3]

2-Amino-4-oxo-*N*-propyl-3,4-dihydropteridine-7-carboxamide (6): Compound **6** was synthesized by the general method, using 37 μL of 1-propylamine and 2 equiv DBU, resulting in 49 mg (87%) of **6**. MP > 300°C. ^1H NMR (400 MHz, DMSO-d_6) δ(ppm)= 11.68 (s, 1H), 8.86 (s, 1H), 8.82 (t, *J* = 6 Hz, 1H), 7.05 (br, 2H), 3.28 (q, *J* = 7.2 Hz, 2H), 1.57 (sext., *J* = 7.2 Hz, 2H), 0.88 (t, *J* = 7.2 Hz, 3H); ^{13}C NMR (100 MHz, DMSO-d_6) δ(ppm)= 163.2, 160.9, 156.3, 154.8, 148.4, 137.1, 132.0, 41.1, 22.8, 11.8; IR (cm^{-1}): 3260, 3130, 2964, 1705, 1644, 1518, 1397; HRMS-ESI (m/z) $[M+H]^+$ calc. for $(C_{10}H_{13}N_6O_2)^+$ 249.1094; found 249.1095

2-Amino-*N*-(2-hydroxypropyl)-4-oxo-3,4-dihydropteridine-7-carboxamide (7): Compound **7** was synthesized by the general method, using 35 μL of 1-amino-2-propanol and 2 equiv DBU, resulting in 51 mg (85%) of **7**. MP > 300°C. ^1H NMR (400 MHz, DMSO-d_6) δ(ppm)= 11.59 (s, 1H), 8.87 (s, 1H), 8.61 (t, *J* = 5.6 Hz, 1H), 7.02 (br, 2H), 4.88 (d, *J* = 4.8 Hz, 1H), 3.82 (quint., *J* = 6.4 Hz, 1H), 3.36 (m, 1H), 3.21 (m, 1H), 1.08 (d, *J* = 6.4 Hz, 3H); ^{13}C NMR (100 MHz, DMSO-d_6) δ(ppm)= 163.0, 160.9, 156.4, 154.9, 148.0, 136.9, 132.2, 65.3, 46.9, 21.6; IR (cm^{-1}): 3400-3100 (broad/overlapping), 2933, 1728, 1679, 1631, 1518, 1230; HRMS-ESI (m/z) $[M+H]^+$ calc. for $(C_{10}H_{13}N_6O_3)^+$ 265.1044; found 265.1043

2-Amino-*N*-(4-aminophenethyl)-4-oxo-3,4-dihydropteridine-7-carboxamide (8): Compound **8** was synthesized by the general method above, using 59 μL 4-(2-aminoethyl)aniline and 2 equiv DBU, which provided 56 mg (76%) of **8**. mp > 300°C. ^{1}H NMR (400 MHz, DMSO-d_6) δ(ppm)= 11.59 (s, 1H), 8.85 (s, 1H), 8.74 (t, *J* = 5.6 Hz, 1H), 7.03 (br, 2H), 6.89 (d, *J* = 8 Hz, 2H), 6.51 (d, *J* = 8 Hz, 2H), 5.46 (br, 2H), 3.47 (q, *J* = 6.8 Hz, 2H), 2.69 (t, *J* = 7.2 Hz, 2H); ^{13}C NMR (100 MHz, DMSO-d_6) δ(ppm)= 162.9, 160.8, 154.9, 148.2, 146.9, 137.0, 132.1, 129.4 (2C), 126.7, 121.2, 114.7 (2C), 41.3, 34.7; IR (cm^{-1}): 3413, 3340, 3195, 1731, 1655, 1530, 1426; HRMS-ESI (m/z) $[M+H]^+$ calc. for $(C_{15}H_{16}N_7O_2)^+$ 326.1360; found 326.1358

2-Amino-*N*-(2-hydroxyethyl)-*N*-methyl-4-oxo-3,4-dihydropteridine-7-carboxamide (9): Compound **9** was synthesized by the general method above, using 37 μL *N*-methylaminoethanol and 2 equiv DBU. This resulted in 43 mg (71%) of **9** which exists as a 2:1 mixture of the *s-cis* and *s-trans* rotamers. MP > 300°C. ^{1}H NMR (400 MHz, DMSO-d_6) δ(ppm) = {two rotamers}[4] 8.41 (s, 1H) {8.45 minor rotamer}, 7.07 (br, 2H) {overlapping minor rotamer}, 4.71 (t, *J* = 5.2 Hz, 1H) {4.84 minor rotamer}, 3.51 (q, *J* = 5.2 Hz, 2H) {3.65 minor rotamer}, 3.41 (t, *J* = 5.6 Hz, 2H) {3.55 minor rotamer}, 3.05 (s, 3H) {3.01 minor rotamer}; ^{13}C NMR (100 MHz, DMSO-d_6) δ(ppm)= {two rotamers} 167.0 {1.66.2 minor rotamer}, 161.2, 156.2, 155.1, 154.1 {153.9 minor rotamer}, 138.3 {137.6 minor rotamer}, 129.5 {129.8 minor rotamer}, 58.8 {58.7 minor rotamer}, 52.5 {50.2 minor rotamer}, 33.6 {37.9 minor rotamer}; IR (cm^{-1}): 3395, 3259, 3126, 2734, 1697, 1613, 1520, 1405, 1234; HRMS-ESI (m/z) $[M+H]^+$ calc. for $(C_{10}H_{13}N_6O_3)^+$ 265.1044; found 265.1044

2-Amino-4-oxo-*N*-(2-(pyridin-2-yl)ethyl)-3,4-dihydropteridine-7-carboxamide (10): Compound **10** was synthesized by the general method, using 54 μL 2-(2-aminoethyl)pyridine and 2 equiv DBU, which provided 46 mg (66%) of **10**. mp > 300°C. ^{1}H NMR (400 MHz, DMSO- d_6) δ(ppm)= 11.63 (s, 1H), 8.95 (t, *J* = 6 Hz, 1H), 8.85 (s, 1H), 8.54 (d, *J* = 4 Hz, 1H), 7.78 (t, *J* = 7.2 Hz, 1H), 7.35 (d, *J* = 7.6 Hz, 1H), 7.29 (t, *J* = 6 Hz, 1H), 7.02 (br, 2H), 3.71 (q, *J* = 6.4 Hz, 2H), 3.07 (t, *J* = 6.8 Hz, 2H); ^{13}C NMR (100 MHz, DMSO-d_6) δ(ppm)= 165.5, 163.2, 160.9, 159.1, 154.8, 148.8, 148.1, 137.9, 137.0, 132.1, 124.0, 122.3, 39.1, 37.0; IR (cm^{-1}): 3126, 2973, 1672, 1609, 1436, 1134, 767; HRMS-ESI (m/z) $[M+H]^+$ calc. for $(C_{14}H_{14}N_7O_2)^+$ 312.1203; found 312.1203

2-Amino-*N*-isopropyl-4-oxo-3,4-dihydropteridine-7-carboxamide (11): Compound **11** was synthesized by the general method, using 40 μL of 2-propylamine and 2 equiv DBU, resulting in 37 mg (66%) of **11**. MP > 300°C. ^{1}H NMR (400 MHz, DMSO-d_6) δ(ppm)= 11.59 (s, 1H), 8.85 (s, 1H), 8.48 (d, *J* = 8 Hz, 1H), 6.98 (br, 2H), 4.14 (oct, *J* = 6.8 Hz, 1H), 1.21 (d, *J* = 6.4 Hz, 6H); ^{13}C NMR (100 MHz, DMSO-d_6) δ(ppm)= 165.5, 162.3, 160.9, 154.8, 148.5, 137.1, 131.9, 41.4, 22.5 (2C); IR (cm^{-1}): 3141, 2974, 1695, 1633, 1515, 1386; HRMS-ESI (m/z) $[M+H]^+$ calc. for $(C_{10}H_{13}N_6O_2)^+$ 249.1094; found 249.1093

2-Amino-4-oxo-*N*-(1-phenylethyl)-3,4-dihydropteridine-7-carboxamide (12): Compound **12** was synthesized by the general method, using 59 μL of α-methyl-benzylamine and 2 equiv DBU, resulting in 42 mg (60%) of **12**. MP > 300°C. ^{1}H NMR (400 MHz, DMSO-d_6) δ(ppm)= 11.65 (s, 1H), 9.07 (d, *J* = 8.4 Hz, 1H), 8.83 (s, 1H), 7.44 (d, *J* = 7.2 Hz, 2H), 7.34 (t, *J* = 7.2 Hz, 2H), 7.25 (t, *J* = 7.2 Hz, 1H), 7.03 (br, 2H), 5.19 (quint, *J* = 7.6 Hz, 1H), 1.54 (d, *J* = 7.2 Hz, 3H); ^{13}C NMR (100 MHz, DMSO-d_6) δ(ppm)= 162.6, 161.0, 156.5, 154.9, 148.4, 144.5, 137.2, 132.1, 128.8 (2C), 127.3, 126.7 (2C), 48.9,

22.3; IR (cm^{-1}): 3405, 3141, 1643, 1515, 1397, 773, 693; HRMS-ESI (m/z) $[M+H]^+$ calc. for $(C_{15}H_{15}N_6O_2)^+$ 311.1251; found 311.1252

(2-Amino-4-oxo-3,4-dihydropteridine-7-carbonyl)alanine (13): Compound **13** was synthesized by the general method above, using 41 mg alanine and 4 equiv DBU, resulting in 31 mg (49%) **13**. MP > 300°C. ^{1}H NMR (400 MHz, DMSO-d_6) δ(ppm)= 12.90 (br, 1H), 11.67 (s, 1H), 8.86 (s, 1H), 8.83 (d, *J* = 8 Hz, 1H), 7.04 (br, 2H), 4.48 (quint, *J* = 7.2 Hz, 1H), 1.45 (d, *J* = 7.2 Hz, 3H); ^{13}C NMR (100 MHz, DMSO-d_6) δ(ppm)= 173.9, 165.6, 162.8, 160.8, 154.9, 147.6, 136.9, 132.4, 48.3, 17.7; HRMS-ESI (m/z) $[M–H]^-$ calc. for $(C_{10}H_9N_6O_4)^-$ 277.0691; found 277.0689. This is consistent with that reported in the literature.[3]

Methyl 5-((2-amino-4-oxo-3,4-dihydropteridine-7-carboxamido)methyl)furan-2-carboxylate (14): Compound **14** was synthesized by the general method above, using 69 mg methyl 5-(aminomethyl)-furan-2-carboxylate[5] and 2 equiv DBU. This resulted in a mixture of the product and 7-carboxy-pterin, which was sonicated in a dilute aqueous solution of $NaHCO_3$ to dissolve the acid and the product was isolated by filtration, resulting in 37 mg (47%) **14** after drying. MP > 300°C. ^{1}H NMR (400 MHz, DMSO-d_6) δ(ppm)= 11.62 (s, 1H), 9.45 (t, *J* = 6 Hz, 1H), 8.88 (s, 1H), 7.24 (d, *J* = 3.6 Hz, 1H), 7.02 (br, 2H), 6.5 (d, *J* = 3.2 Hz, 1H), 4.56 (d, *J* = 6 Hz, 2H), 3.79 (s, 3H); ^{13}C NMR (100 MHz, DMSO-d_6) δ(ppm)= 163.5, 160.8, 158.7, 157.4, 156.2, 154.8, 147.9, 143.2, 137.2, 132.3, 119.8, 110.0, 52.1, 36.7; IR (cm^{-1}): 3126, 1681, 1518, 1338, 1204, 1016, 761 HRMS-ESI (m/z) $[M+H]^+$ calc. for $(C_{14}H_{13}N_6O_5)^+$ 345.0942; found 345.0942

2-Amino-7-(morpholine-4-carbonyl)pteridin-4(3*H*)-one (15): Compound **15** was synthesized by the general method above, using 40 μL morpholine and 2 equiv DBU. Initially this resulted in a 1:1 mixture of the product and 7-carboxy-pterin, which was sonicated in a dilute aqueous solution of $NaHCO_3$ to dissolve the carboxylate, and the product was collected by filtration, resulting in 28 mg (44%) **15** after drying. MP > 300°C. ^{1}H NMR (400 MHz, DMSO-d_6) δ(ppm)= 11.64 (s, 1H), 8.49 (s, 1H), 7.07 (br, 2H), 3.68 (m, 4H), 3.58 (m, 2H), 3.49 (m, 2H); ^{13}C NMR (100 MHz, DMSO-d_6) δ(ppm)= 164.9, 161.2, 156.3, 155.2, 152.8, 137.9, 130.1, 66.7, 66.4, 47.4, 42.5; IR (cm^{-1}): 3438, 3336, 3084, 2986, 2934, 1720, 1632, 1595, 1204; HRMS-ESI (m/z) $[M+H]^+$ calc. for $(C_{11}H_{13}N_6O_3)^+$ 277.1044; found 277.1043

2-Amino-*N*-benzyl-*N*-methyl-4-oxo-3,4-dihydropteridine-7-carboxamide (16): Synthesis of **16** required a modified method, where 0.3 mL (10 equiv) of *N*-methyl-benzylamine was added to the solition of 50 mg 7CMP (**1**) in 1 mL MeOH and 2 equiv of DBU. The reaction vessel was sealed and heated to 130 °C for 3 h. Work-up proceeded as described in the general method, providing 44 mg (63%) of **16**, existing as an equal mixture of the *s-cis* and *s-trans* rotamers. MP > 300°C. ^{1}H NMR (400 MHz, DMSO-d_6) δ(ppm)= {two rotamers}[4] 11.57 {overlapping rotamer} (s, 1H), 8.53 {8.49} (s, 1H), 7.42-7.27 {overlapping rotamer} (m, 5H), 7.03 {overlapping rotamer} (br, 2H), 4.72 {4.58} (s, 2H), 2.91 {2.90} (s, 3H); ^{13}C NMR (100 MHz, DMSO-d_6) δ(ppm)= {two rotamers} 166.7 {166.5}, 161.1 {overlapping}, 155.2 {overlapping}, 153.6 {153.5}, 137.7 {137.6}, 137.2 {137.1}, 130.1 {130.0}, 129.1(2C) {129.0}, 128.9 {128.8}, 128.1(2C) {127.9}, 127.8 {overlapping}, 53.7 {50.4}, 36.6 {32.9}; IR (cm^{-1}): 3150, 2744, 1696, 1658, 1512, 1407, 1334, 777, 637; HRMS-ESI (m/z) $[M+H]^+$ calc. for $(C_{15}H_{15}N_6O_2)^+$ 311.1251; found 311.1251

Pseudo-first order kinetics NMR data collection:

Kinetics for the DBU-amidation was followed by ^{1}H NMR for a representative set of amines. Pseudo-first order conditions were used, whereby the amine was in large excess and its change in concentration was negligible.[5] Therefore, the rate could be viewed as:

Rate = k_{obs}[7-CMP], where $k_{obs} = k[\text{Amine}]_o$

Therefore the integrated rate law becomes $\ln[\text{7-CMP}]_t = \ln[\text{7-CMP}]_o + k_{obs}t$, and plotting ln[7-CMP] vs t gives a slope of k_{obs}. The k value for the 2nd order rate is thus determined by dividing k_{obs} by the initial amine concentration.[5]

A typical ^{1}H NMR experiment begins by suspending 7-CMP in anhydrous methanol-d_4, followed by the addition of DBU and a large excess of the amine to provide a homogeneous solution. This solution is then transferred to an NMR tube, and a spectrum is collected every 60 min for 4–8 h. For faster reactions, data was collected every 20 min for the first hour. As glycine gave a particularly rapid reaction, data was collected every 10 min for the first 2 h. The disappearance of the aromatic C_6-H proton of 7-CMP (appears at 8.8 ppm in methanol-d_4) was coupled with the growth of the aromatic C_6-H proton for the amide product (typically appears between 8.91–8.95 ppm in methanol-d_4). Ratios of integrals were converted to %(7-CMP) and %(product), which was in turn converted to $[\text{7-CMP}]_t$.

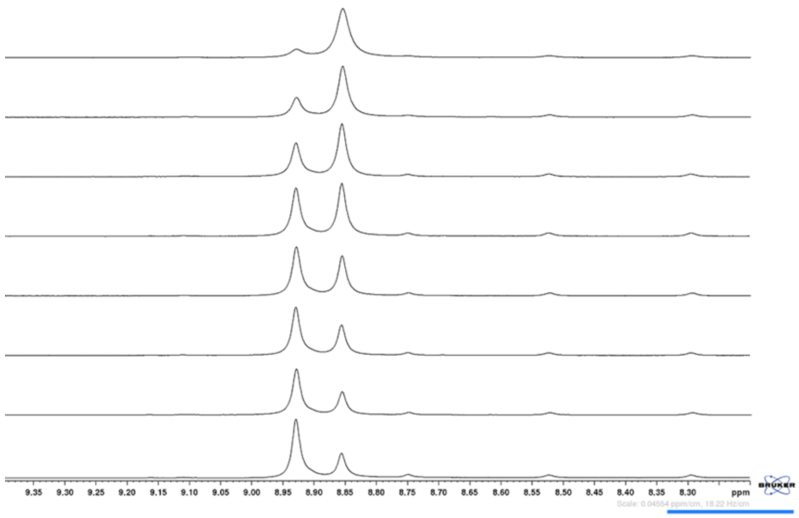

Figure S1: Representative NMR data, showing the shift in the pterin C_6-H signal as 7-CMP reacts with glycine from t = 5 min (top) to t = 75 min (bottom) in methanol-d_4.

Copies of ^{1}H and ^{13}C spectra:

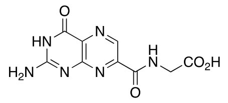

2 (in DMSO-d_6)

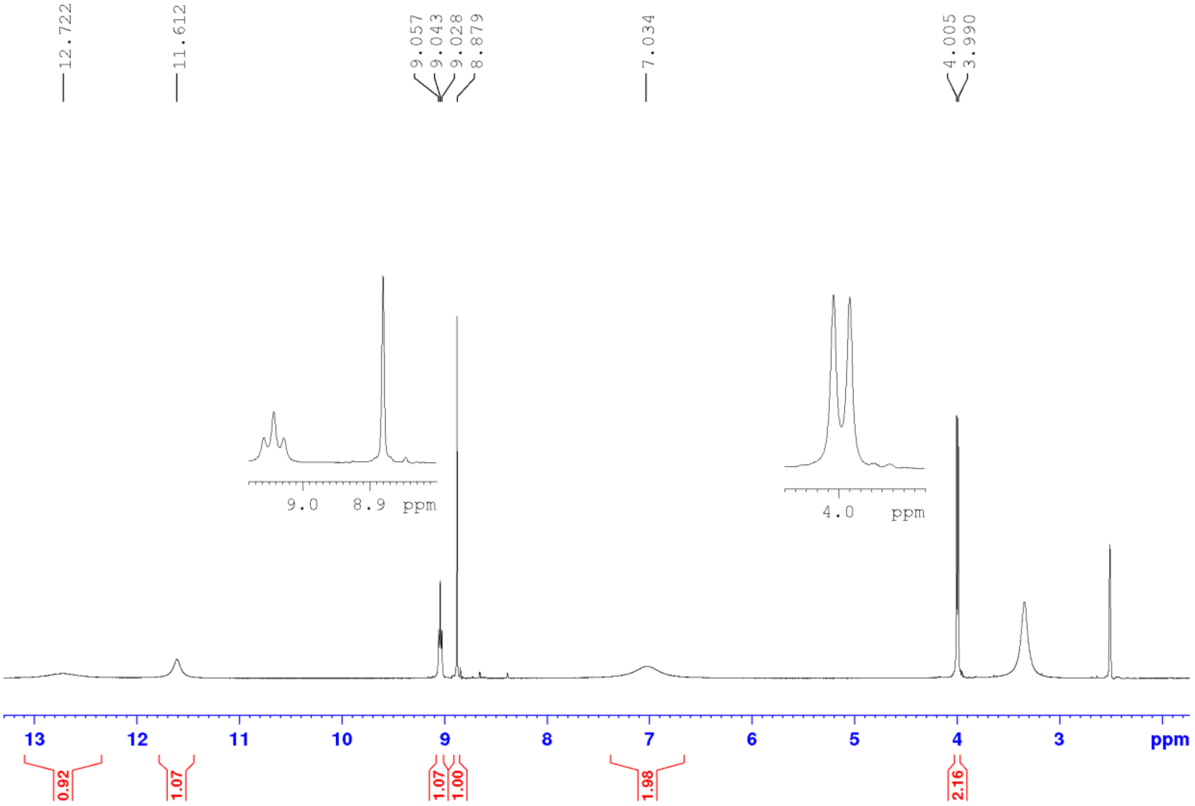

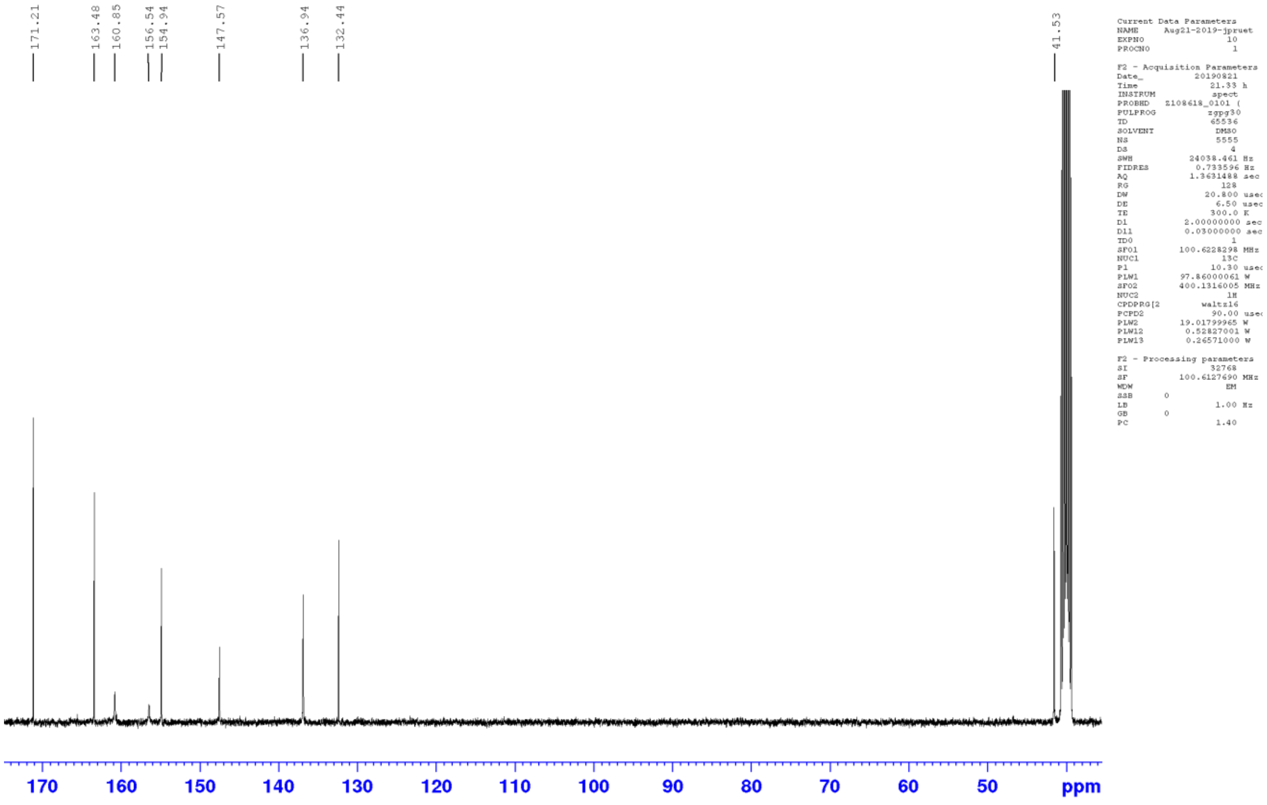

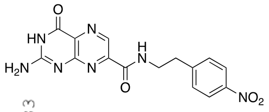
O
HN
N
H
N
H2N
N
N
O
NO2

3 (in DMSO-d_6)

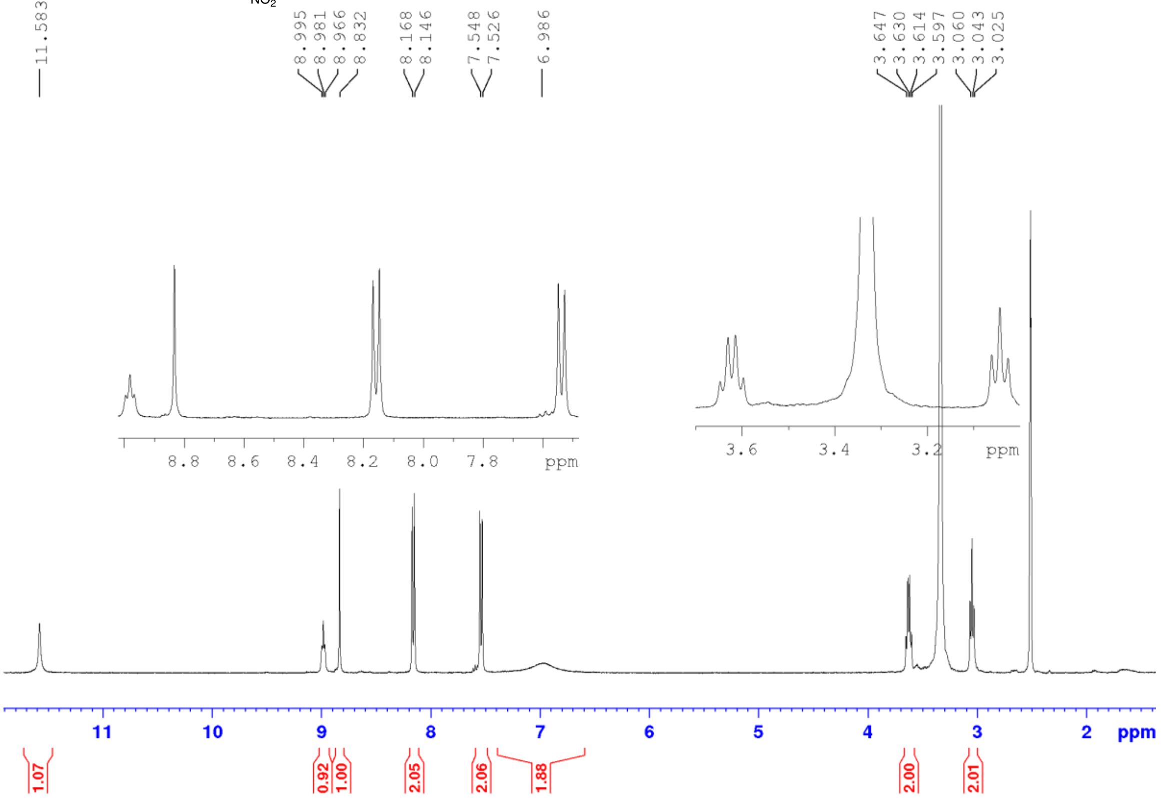
11.583
8.995
8.981
8.966
8.832
8.168
8.146
7.548
7.526
6.986
3.647
3.630
3.614
3.597
3.060
3.043
3.025
8.8 8.6 8.4 8.2 8.0 7.8 ppm
3.6 3.4 3.2 ppm
11 10 9 8 7 6 5 4 3 2 ppm
1.07
0.92
1.00
2.05
2.06
1.88
2.00
2.01

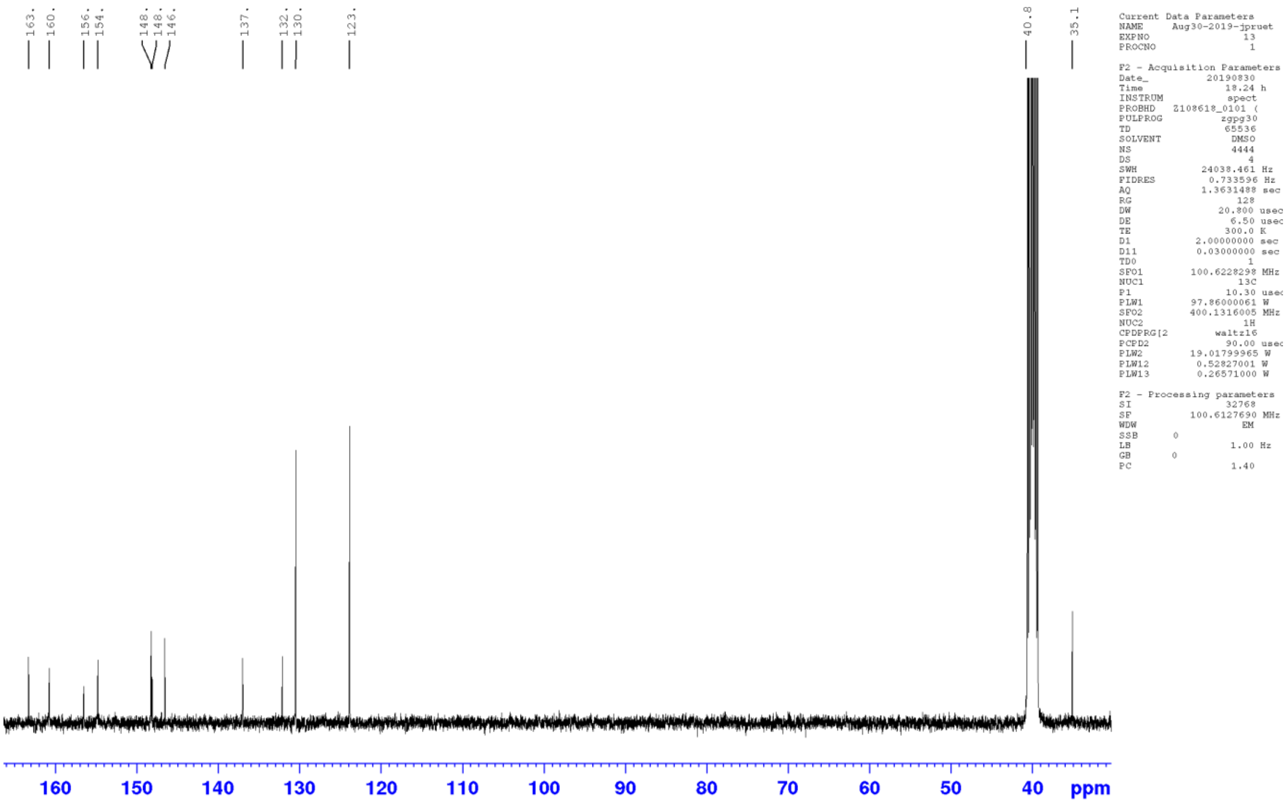
163.
160.
156.
154.
148.
148.
146.
137.
132.
130.
123.
40.8
35.1
Current Data Parameters
NAME Aug30-2019-jpruet
EXPNO 13
PROCNO 1
F2 - Acquisition Parameters
Date_ 20190830
Time 18.24 h
INSTRUM spect
PROBHD Z108618_0101 (
PULPROG zgpg30
TD 65536
SOLVENT DMSO
NS 4444
DS 4
SWH 24038.461 Hz
FIDRES 0.733596 Hz
AQ 1.3631488 sec
RG 128
DW 20.800 usec
DE 6.50 usec
TE 300.0 K
D1 2.00000000 sec
D11 0.03000000 sec
TD0 1
SFO1 100.6228298 MHz
NUC1 13C
P1 10.30 usec
PLW1 97.86000061 W
SFO2 400.1316005 MHz
NUC2 1H
CPDPRG[2 waltz16
PCPD2 90.00 usec
PLW2 19.01799965 W
PLW12 0.52827001 W
PLW13 0.26571000 W
F2 - Processing parameters
SI 32768
SF 100.6127690 MHz
WDW EM
SSB 0
LB 1.00 Hz
GB 0
PC 1.40
160 150 140 130 120 110 100 90 80 70 60 50 40 ppm

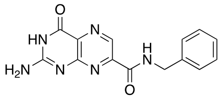
O
HN
N
H
N
H2N
N
N
O

4 (in DMSO-d_6)

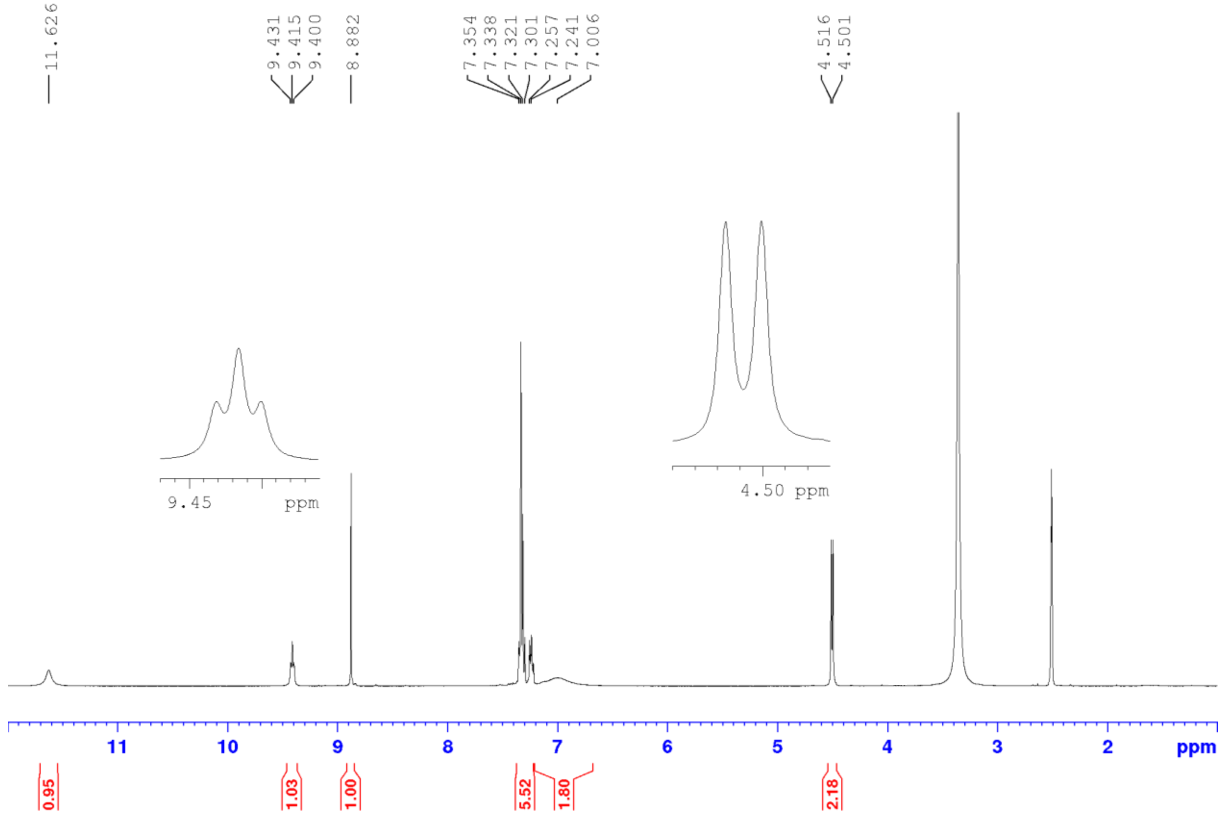
11.626
9.431
9.415
9.400
8.882
7.354
7.338
7.321
7.301
7.257
7.241
7.006
4.516
4.501
9.45
ppm
4.50 ppm
11
10
9
8
7
6
5
4
3
2
ppm
0.95
1.03
1.00
5.52
1.80
2.18

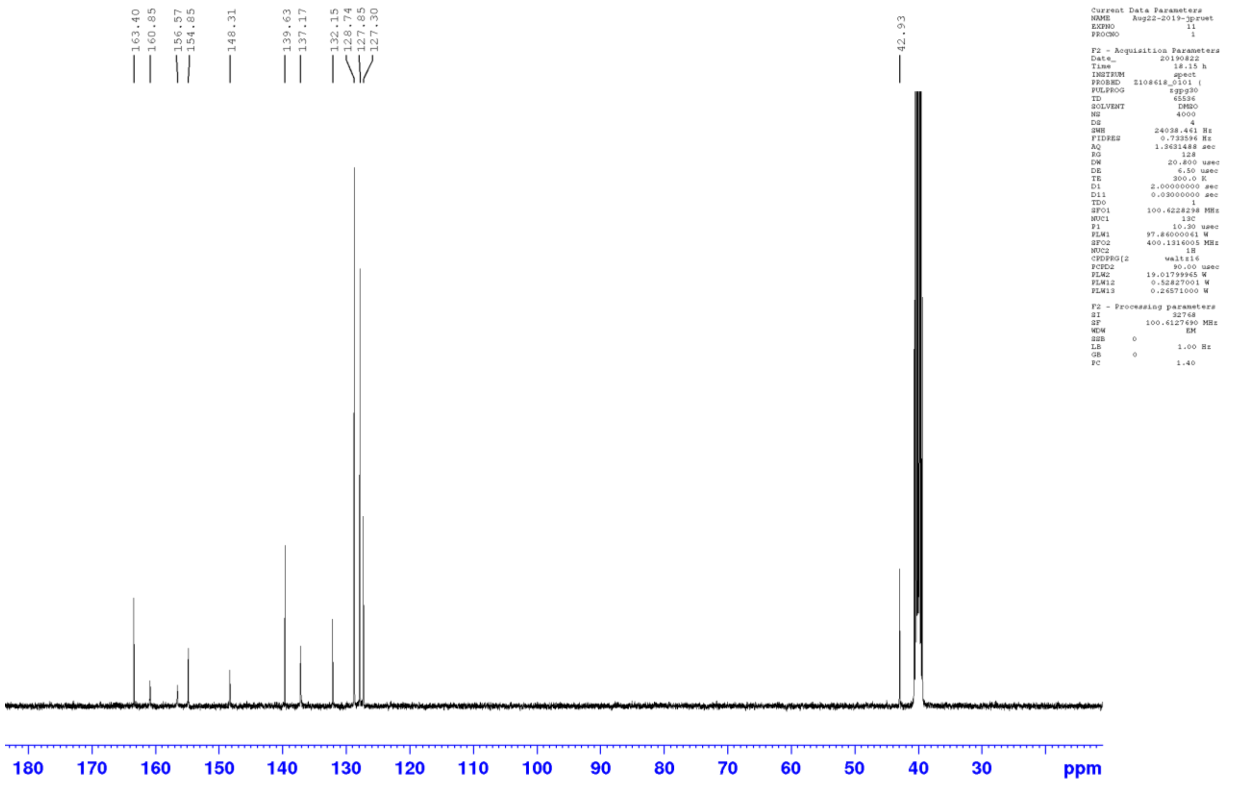
163.40
160.85
156.57
154.85
148.31
139.63
137.17
132.15
128.74
127.85
127.30
42.93
Current Data Parameters
NAME Aug22-2019-jpruet
EXPNO 11
PROCNO 1
F2 - Acquisition Parameters
Date_ 20190822
Time 18.15 h
INSTRUM spect
PROBHD Z108618_0101 (
PULPROG zgpg30
TD 65536
SOLVENT DMSO
NS 4000
DS 4
SWH 24038.461 Hz
FIDRES 0.733596 Hz
AQ 1.3631488 sec
RG 128
DW 20.800 usec
DE 6.50 usec
TE 300.0 K
D1 2.00000000 sec
D11 0.03000000 sec
TD0 1
SFO1 100.6228298 MHz
NUC1 13C
P1 10.30 usec
PLW1 97.86000061 W
SFO2 400.1316005 MHz
NUC2 1H
CPDPRG[2 waltz16
PCPD2 90.00 usec
PLW2 19.01799965 W
PLW12 0.52827001 W
PLW13 0.26571000 W
F2 - Processing parameters
SI 32768
SF 100.6127690 MHz
WDW EM
SSB 0
LB 1.00 Hz
GB 0
PC 1.40
180
170
160
150
140
130
120
110
100
90
80
70
60
50
40
30
ppm

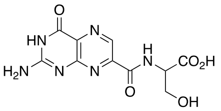

5 (in DMSO-d_6)

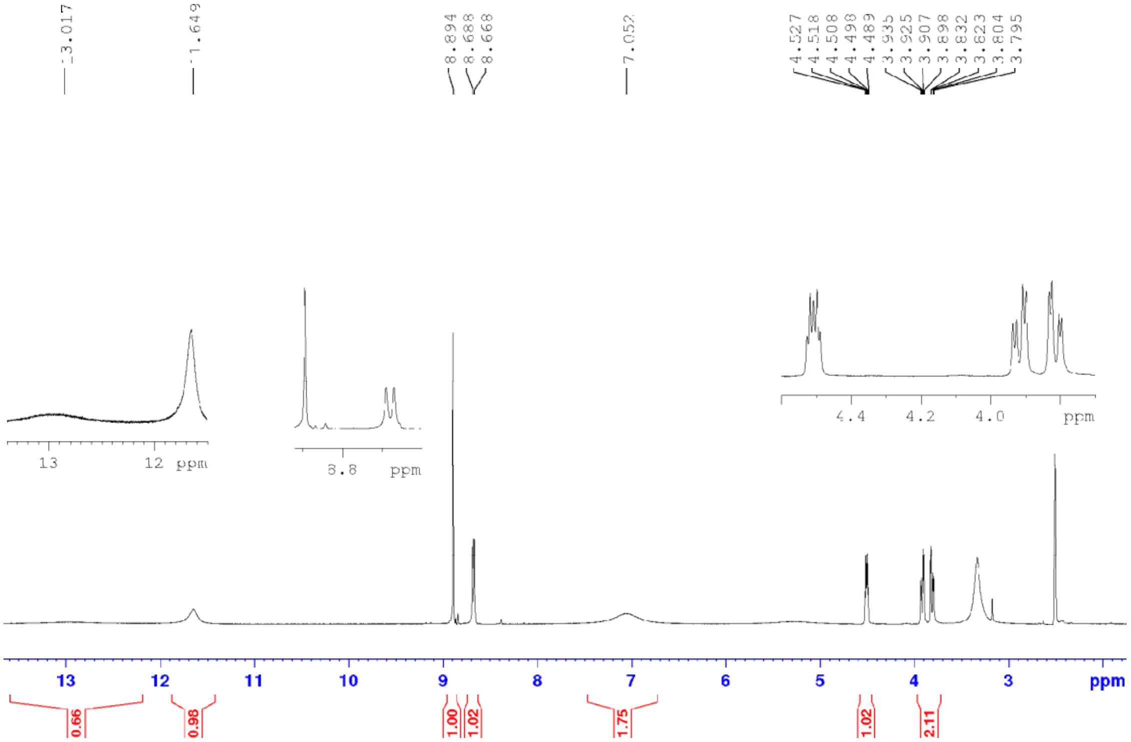

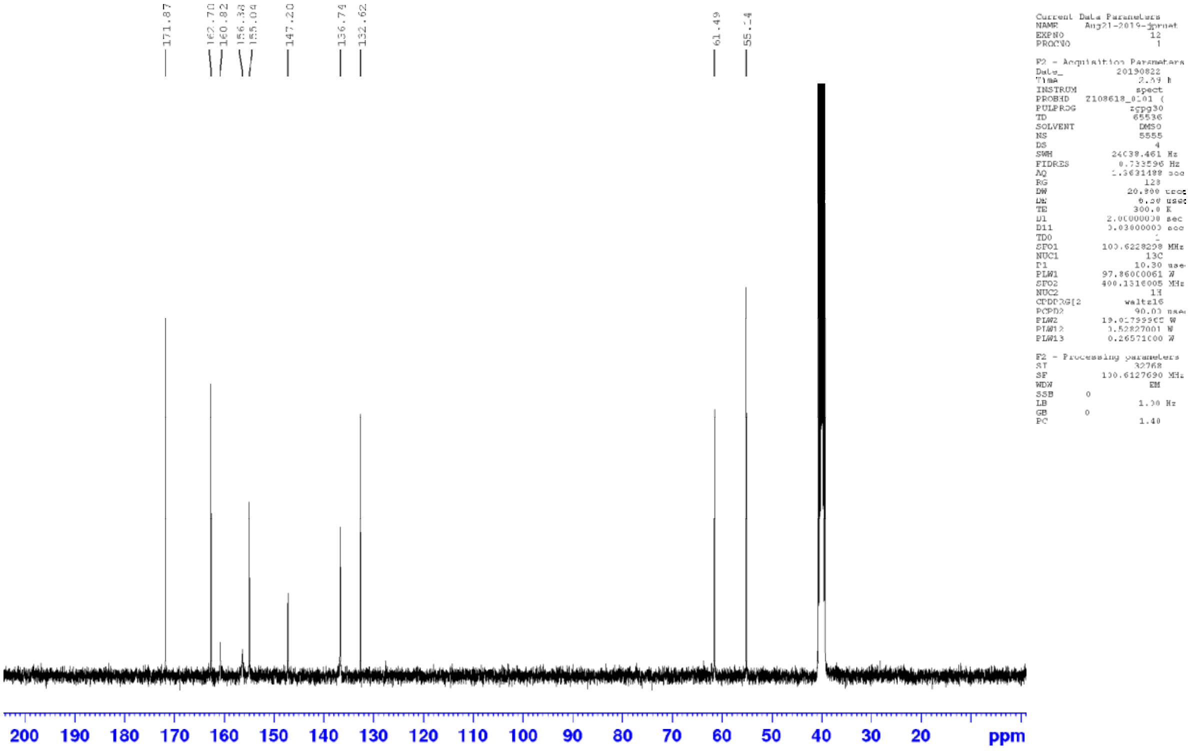

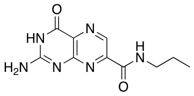
O
HN
N
H2N
N
N
H
N
O

6 (in DMSO-*d*$_6$)

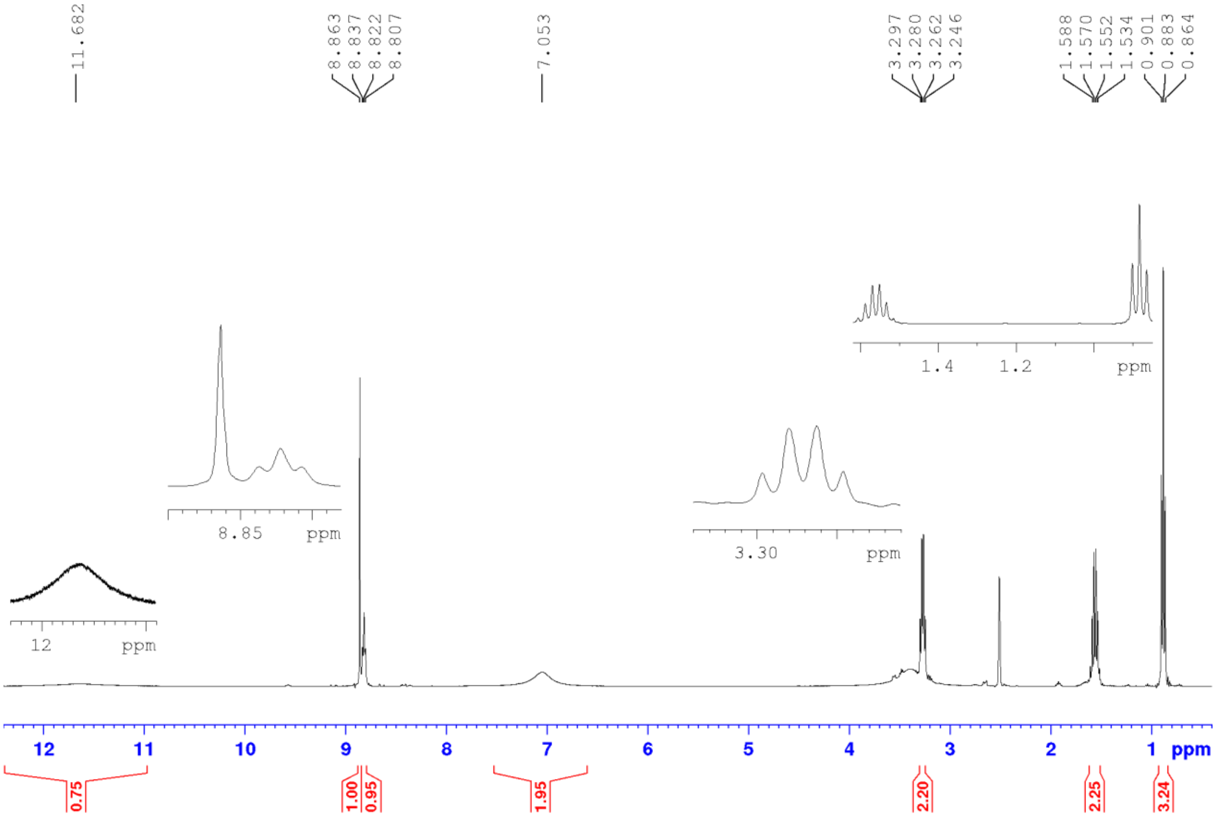
11.682
8.863
8.837
8.822
8.807
7.053
3.297
3.280
3.262
3.246
1.588
1.570
1.552
1.534
0.901
0.883
0.864
12 ppm
8.85 ppm
3.30 ppm
1.4 1.2 ppm
12 11 10 9 8 7 6 5 4 3 2 1 ppm
0.75
1.00
0.95
1.95
2.20
2.25
3.24

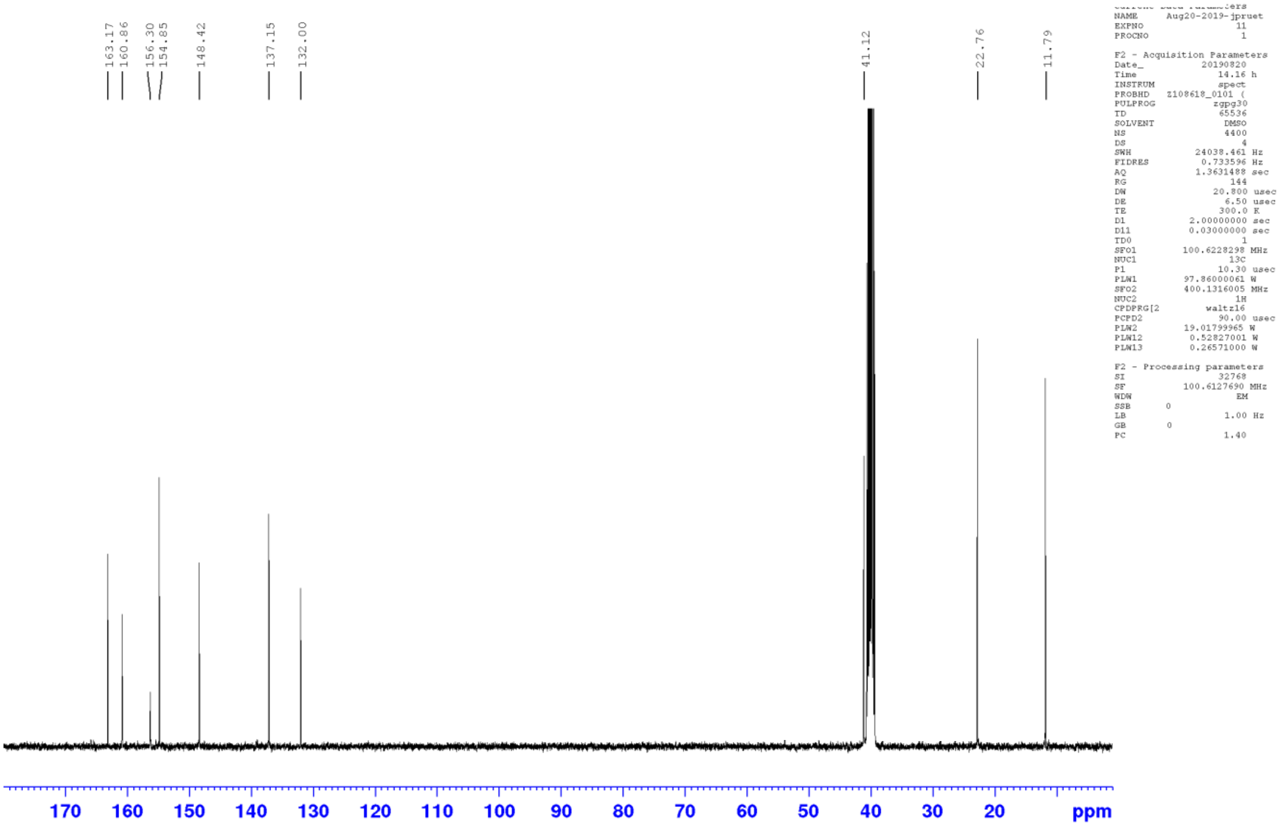
163.17
160.86
156.30
154.85
148.42
137.15
132.00
41.12
22.76
11.79
NAME Aug20-2019-jpruet
EXPNO 11
PROCNO 1
F2 - Acquisition Parameters
Date_ 20190820
Time 14.16 h
INSTRUM spect
PULPROG zgpg30
TD 65536
SOLVENT DMSO
NS 4400
DS 4
SWH 24038.461 Hz
FIDRES 0.733596 Hz
AQ 1.3631488 sec
RG 144
DW 20.800 usec
DE 6.50 usec
TE 300.0 K
D1 2.00000000 sec
D11 0.03000000 sec
TD0 1
SFO1 100.6228298 MHz
NUC1 13C
P1 10.30 usec
PLW1 97.86000061 W
SFO2 400.1316005 MHz
NUC2 1H
CPDPRG[2 waltz16
PCPD2 90.00 usec
PLW2 19.01799965 W
PLW12 0.52827001 W
PLW13 0.26571000 W
F2 - Processing parameters
SI 32768
SF 100.6127690 MHz
WDW EM
SSB 0
LB 1.00 Hz
GB 0
PC 1.40
170 160 150 140 130 120 110 100 90 80 70 60 50 40 30 20 ppm

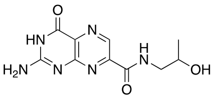

7 (in DMSO-d_6)

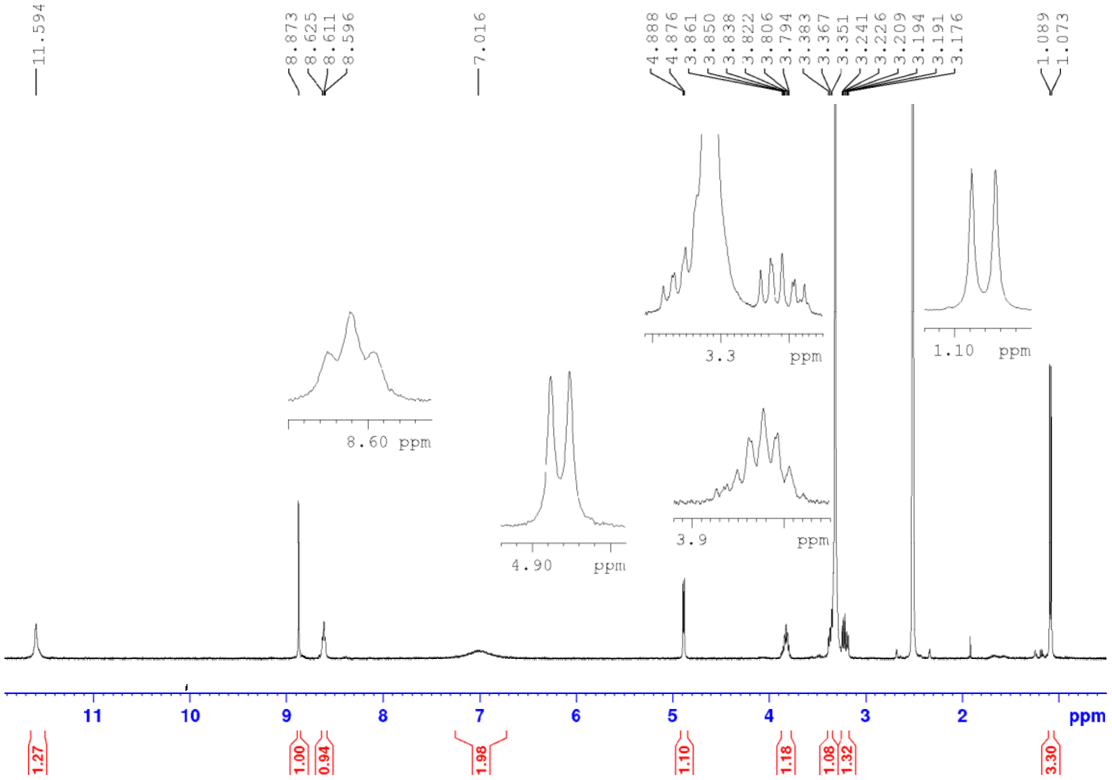
11.594
8.873
8.625
8.611
8.596
7.016
4.888
4.876
3.861
3.850
3.838
3.822
3.806
3.794
3.383
3.367
3.351
3.241
3.226
3.209
3.194
3.191
3.176
1.089
1.073
8.60 ppm
4.90 ppm
3.3 ppm
3.9 ppm
1.10 ppm
11
10
9
8
7
6
5
4
3
2
ppm
1.27
1.00
0.94
1.98
1.10
1.18
1.08
1.32
3.30

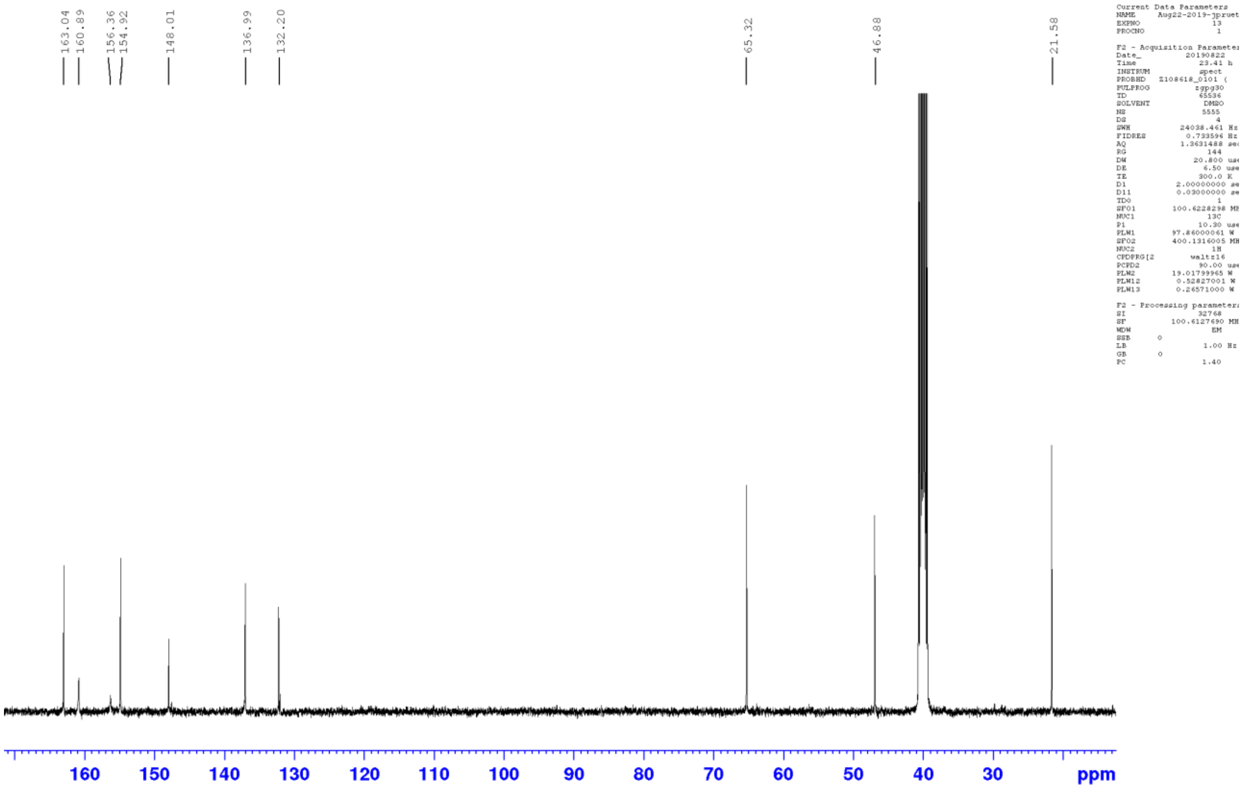
163.04
160.89
156.36
154.92
148.01
136.99
132.20
65.32
46.88
21.58
160
150
140
130
120
110
100
90
80
70
60
50
40
30
ppm

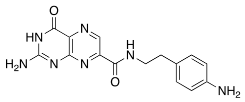

O
HN
N
H
N
H_2N
N
N
O
NH_2

8 (in DMSO-*d*6)

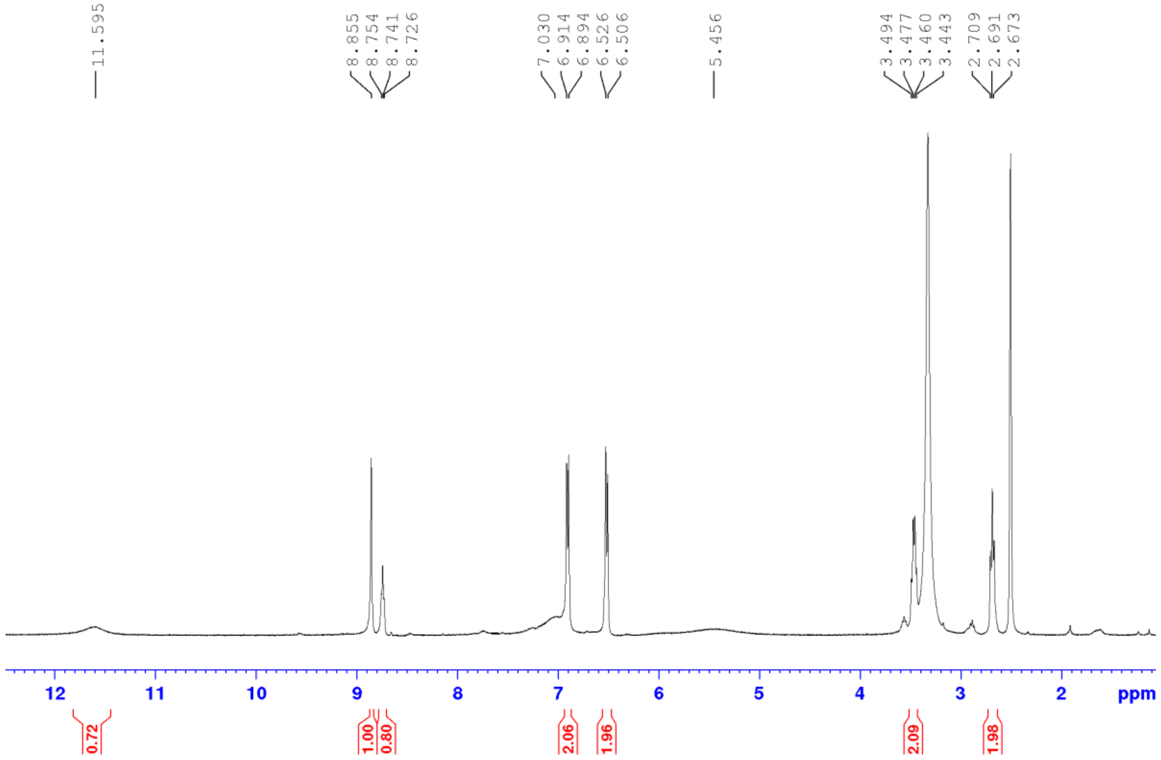

11.595
8.855
8.754
8.741
8.726
7.030
6.914
6.894
6.526
6.506
5.456
3.494
3.477
3.460
3.443
2.709
2.691
2.673
12
11
10
9
8
7
6
5
4
3
2
ppm
0.72
1.00
0.80
2.06
1.96
2.09
1.98

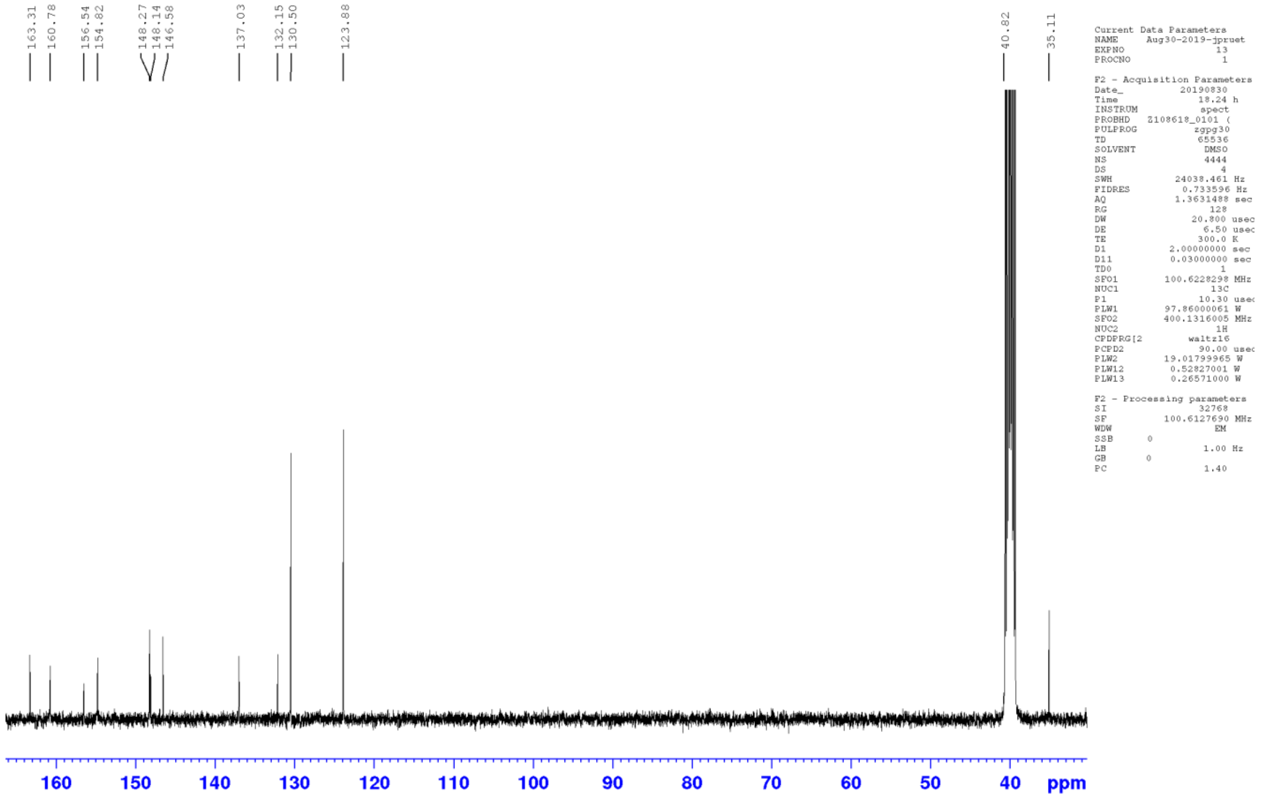

163.31
160.78
156.54
154.82
148.27
148.14
146.58
137.03
132.15
130.50
123.88
40.82
35.11
160
150
140
130
120
110
100
90
80
70
60
50
40
ppm
Current Data Parameters
NAME Aug30-2019-jpruet
EXPNO 13
PROCNO 1
F2 - Acquisition Parameters
Date_ 20190830
Time 18.24 h
INSTRUM spect
PROBHD Z108618_0101 (
PULPROG zgpg30
TD 65536
SOLVENT DMSO
NS 4444
DS 4
SWH 24038.461 Hz
FIDRES 0.733596 Hz
AQ 1.3631488 sec
RG 128
DW 20.800 usec
DE 6.50 usec
TE 300.0 K
D1 2.00000000 sec
D11 0.03000000 sec
TD0 1
SFO1 100.6228298 MHz
NUC1 13C
P1 10.30 usec
PLW1 97.86000061 W
SFO2 400.1316005 MHz
NUC2 1H
CPDPRG[2 waltz16
PCPD2 90.00 usec
PLW2 19.01799965 W
PLW12 0.52827001 W
PLW13 0.26571000 W
F2 - Processing parameters
SI 32768
SF 100.6127690 MHz
WDW EM
SSB 0
LB 1.00 Hz
GB 0
PC 1.40

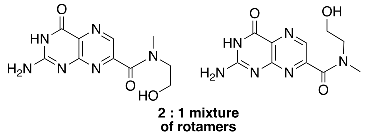
2 : 1 mixture
of rotamers

9 (in DMSO-d_6)

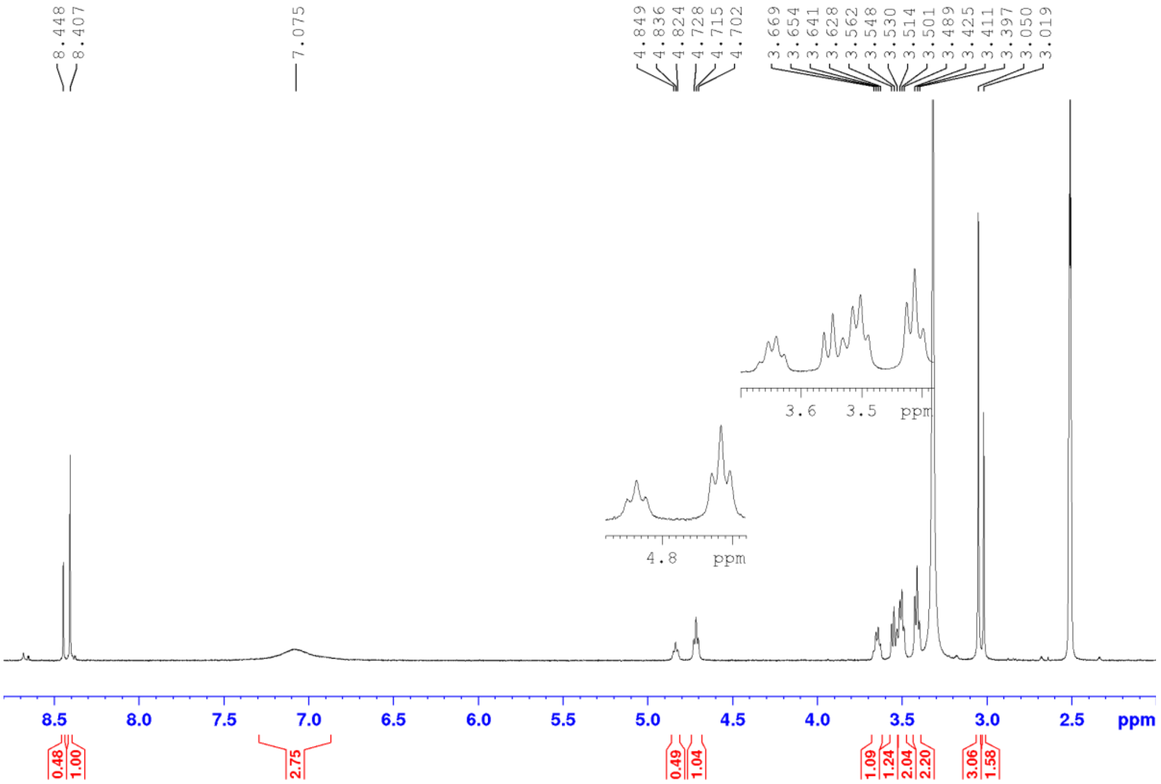
8.448
8.407
7.075
4.849
4.836
4.824
4.728
4.715
4.702
3.669
3.654
3.641
3.628
3.562
3.548
3.530
3.514
3.501
3.489
3.425
3.411
3.397
3.050
3.019
0.48
1.00
2.75
0.49
1.04
1.09
1.24
2.04
2.20
3.06
1.58
ppm

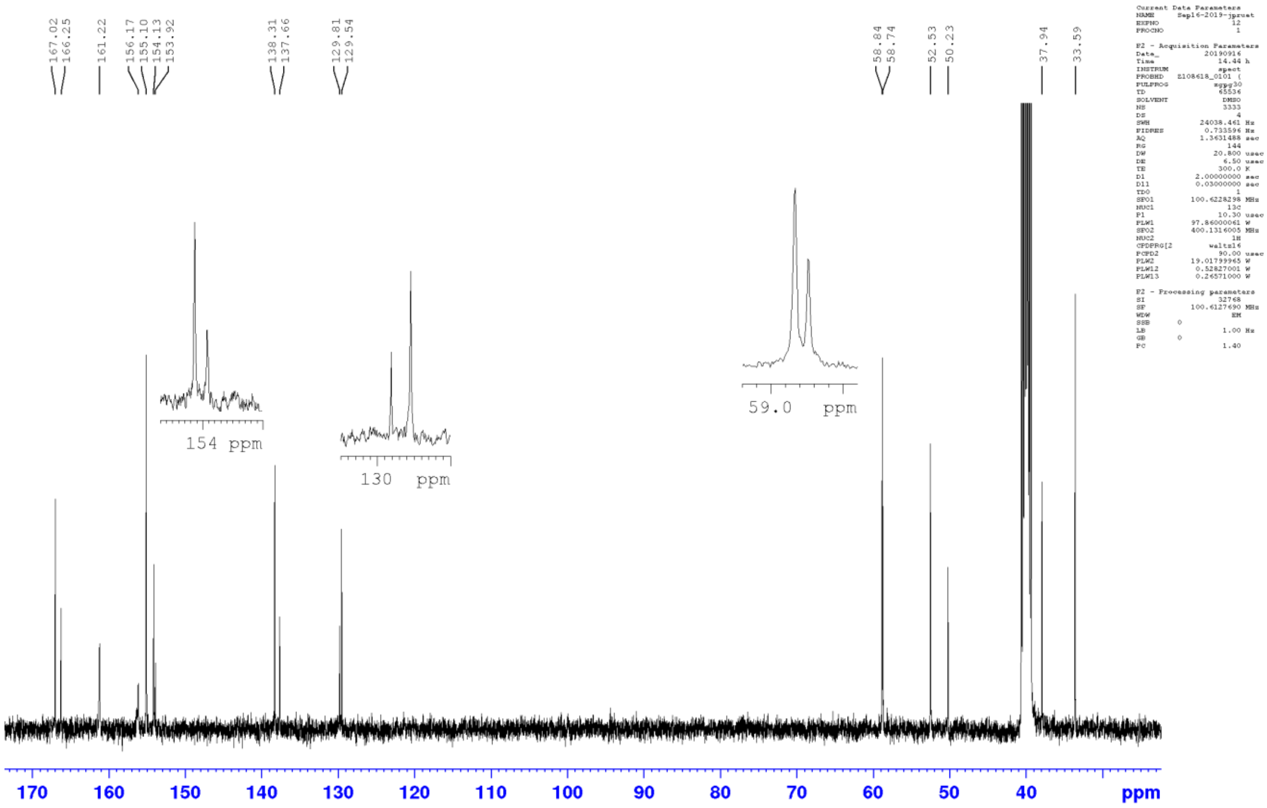
167.02
166.25
161.22
156.17
155.10
154.13
153.92
138.31
137.66
129.81
129.54
58.84
58.74
52.53
50.23
37.94
33.59
154 ppm
130 ppm
59.0 ppm
ppm

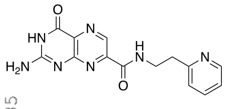

10 (in DMSO-d_6)

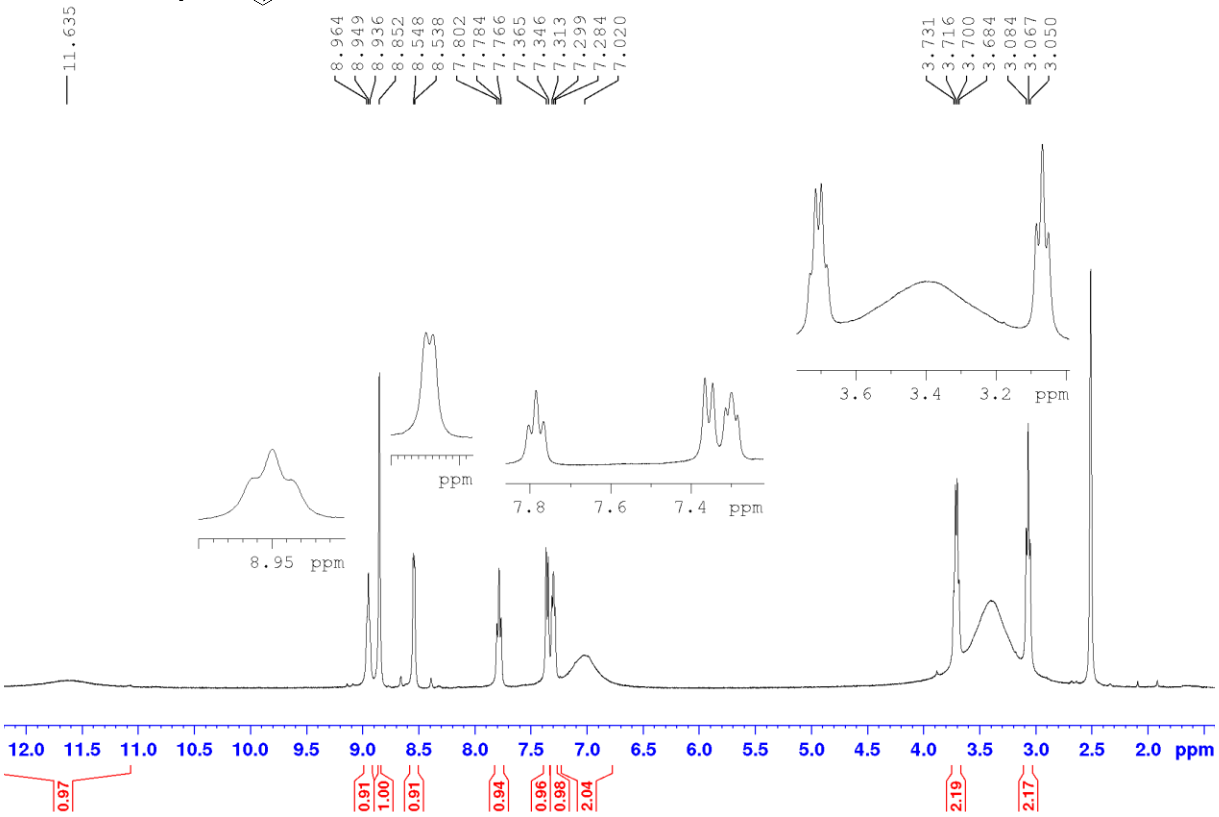

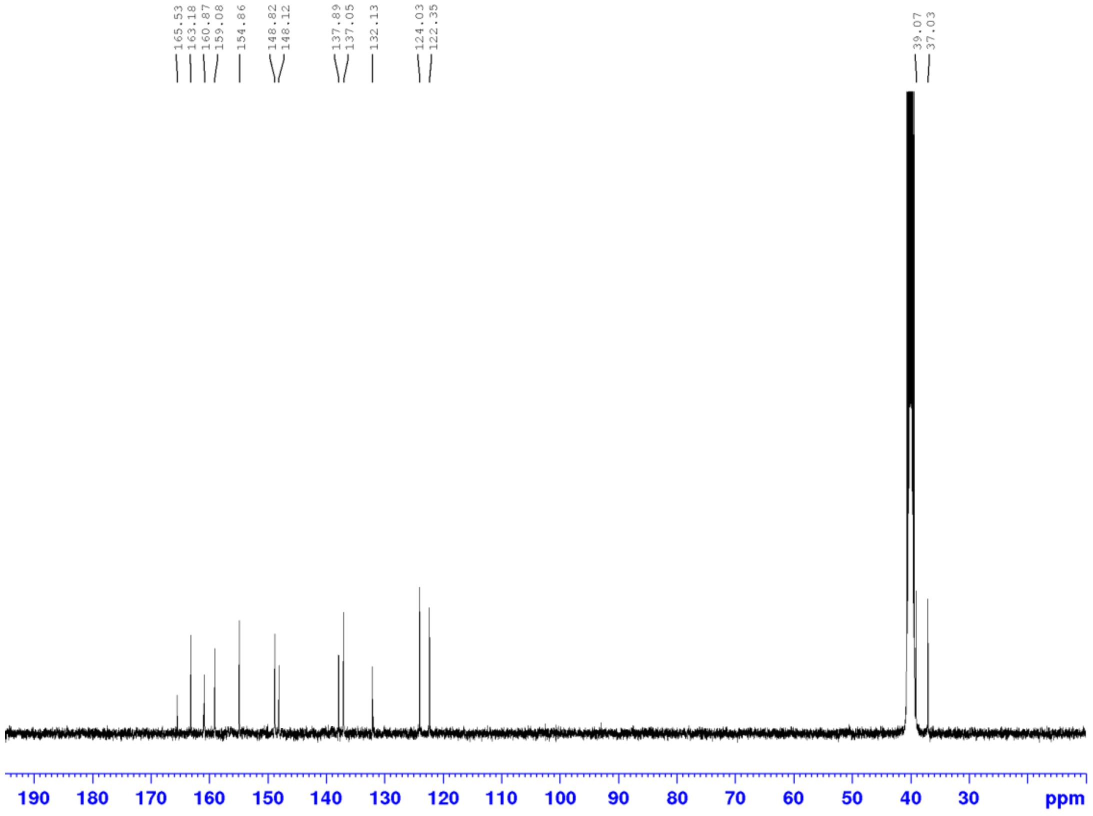


```
Current Data Parameters
NAME      Sep13-2019-jpruet
EXPNO                    11
PROCNO                    1

F2 - Acquisition Parameters
Date_              20190913
Time                18.30 h
INSTRUM               spect
PROBHD   Z108618_0101 (
PULPROG              zgpg30
TD                    65536
SOLVENT                DMSO
NS                     5555
DS                        4
SWH            24038.461 Hz
FIDRES          0.733596 Hz
AQ             1.3631488 sec
RG                      128
DW                20.800 use
DE                  6.50 use
TE                  300.0 K
D1            2.00000000 sec
D11           0.03000000 sec
TD0                       1
SFO1         100.6228298 MHz
NUC1                    13C
P1                 10.30 use
PLW1         97.86000061 W
SFO2         400.1316005 MHz
NUC2                     1H
CPDPRG[2            waltz16
PCPD2              90.00 use
PLW2         19.01799965 W
PLW12         0.52827001 W
PLW13         0.26571000 W

F2 - Processing parameters
SI                    32768
SF           100.6127690 MHz
WDW                      EM
SSB      0
LB                  1.00 Hz
GB       0
PC                     1.40
```

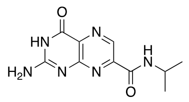
O
HN
N
N
H2N
N
N
H
N
O

11 (in DMSO-*d*$_6$)

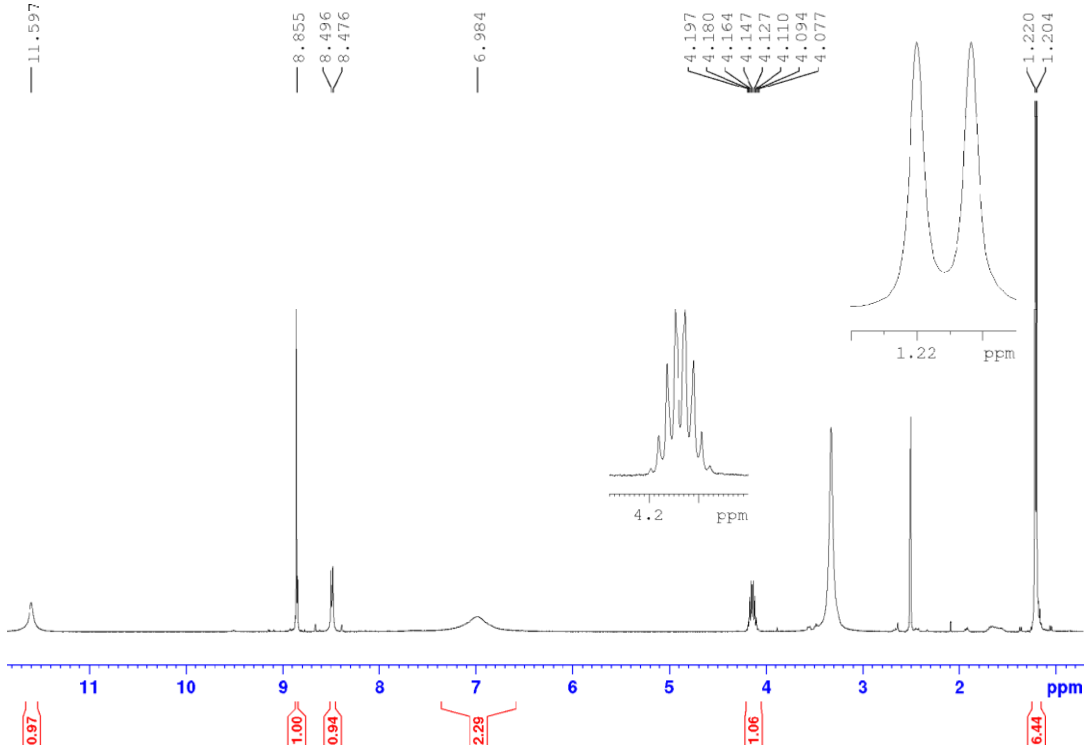
11.597
8.855
8.496
8.476
6.984
4.197
4.180
4.164
4.147
4.127
4.110
4.094
4.077
1.220
1.204
1.22
ppm
4.2
ppm
11
10
9
8
7
6
5
4
3
2
ppm
0.97
1.00
0.94
2.29
1.06
6.44

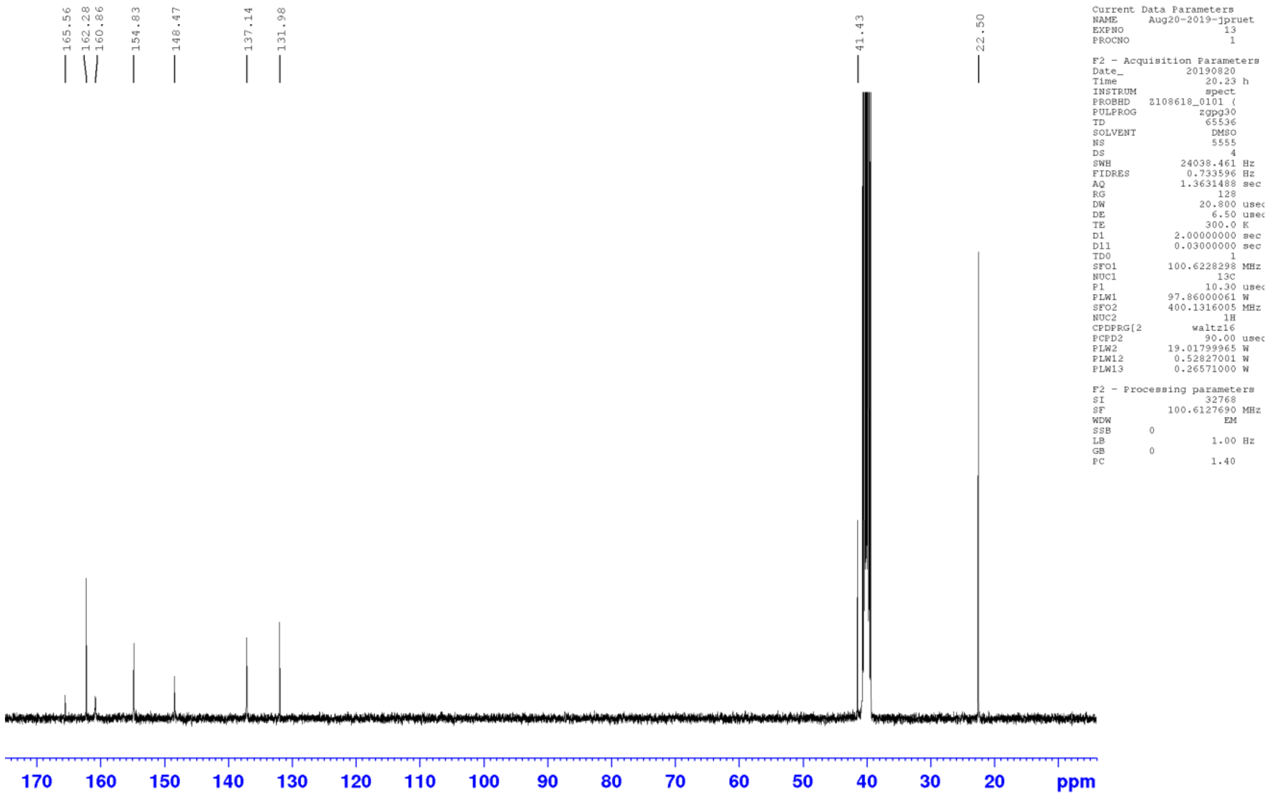
165.56
162.28
160.86
154.83
148.47
137.14
131.98
41.43
22.50
Current Data Parameters
NAME Aug20-2019-jpruet
EXPNO 13
PROCNO 1
F2 - Acquisition Parameters
Date_ 20190820
Time 20.23 h
INSTRUM spect
PROBHD Z108618_0101 (
PULPROG zgpg30
TD 65536
SOLVENT DMSO
NS 5555
DS 4
SWH 24038.461 Hz
FIDRES 0.733596 Hz
AQ 1.3631488 sec
RG 128
DW 20.800 use
DE 6.50 use
TE 300.0 K
D1 2.00000000 sec
D11 0.03000000 sec
TD0 1
SFO1 100.6228298 MHz
NUC1 13C
P1 10.30 use
PLW1 97.86000061 W
SFO2 400.1316005 MHz
NUC2 1H
CPDPRG[2 waltz16
PCPD2 90.00 use
PLW2 19.01799965 W
PLW12 0.52827001 W
PLW13 0.26571000 W
F2 - Processing parameters
SI 32768
SF 100.6127690 MHz
WDW EM
SSB 0
LB 1.00 Hz
GB 0
PC 1.40
170
160
150
140
130
120
110
100
90
80
70
60
50
40
30
20
ppm

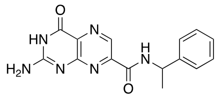

O
HN
N
H
N
H_2N
N
N
O

12 (in DMSO-d_6)

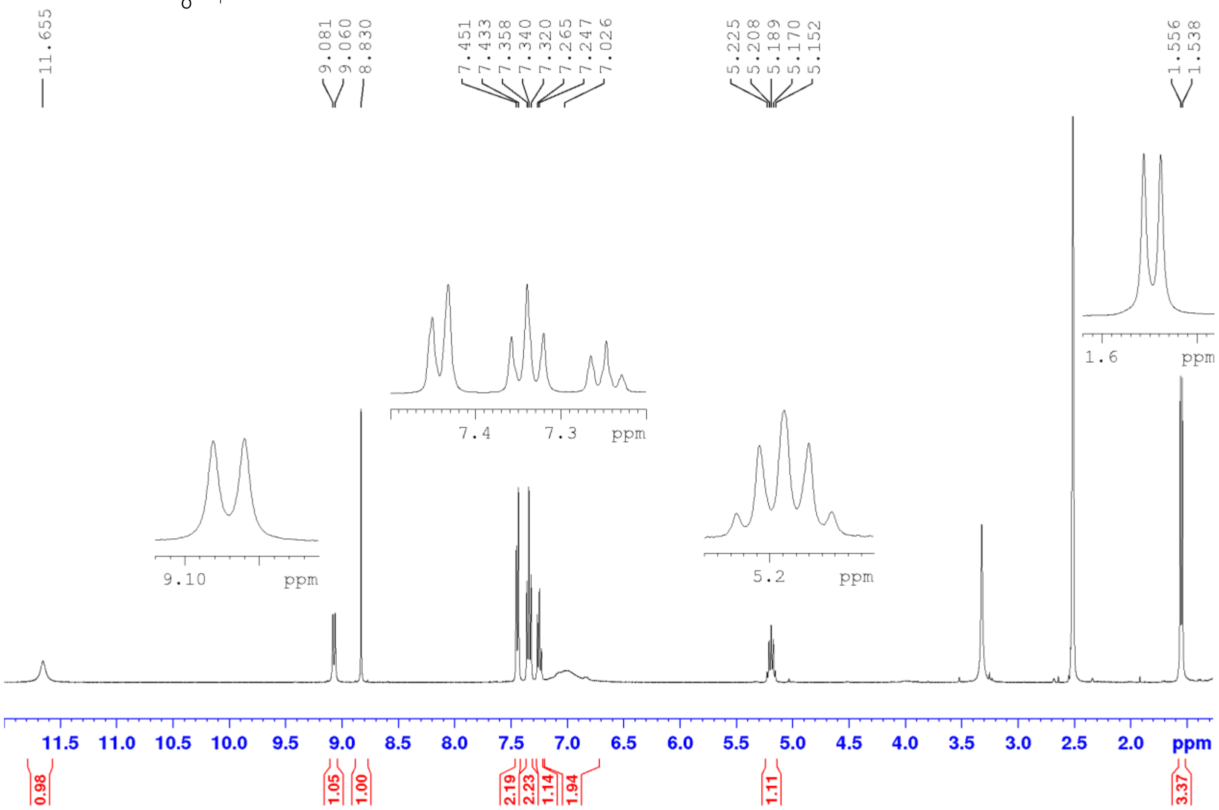

11.655
9.081
9.060
8.830
7.451
7.433
7.358
7.340
7.320
7.265
7.247
7.026
5.225
5.208
5.189
5.170
5.152
1.556
1.538
9.10
ppm
7.4
7.3
ppm
5.2
ppm
1.6
ppm
11.5 11.0 10.5 10.0 9.5 9.0 8.5 8.0 7.5 7.0 6.5 6.0 5.5 5.0 4.5 4.0 3.5 3.0 2.5 2.0 ppm
0.98
1.05
1.00
2.19
2.23
1.14
1.94
1.11
3.37

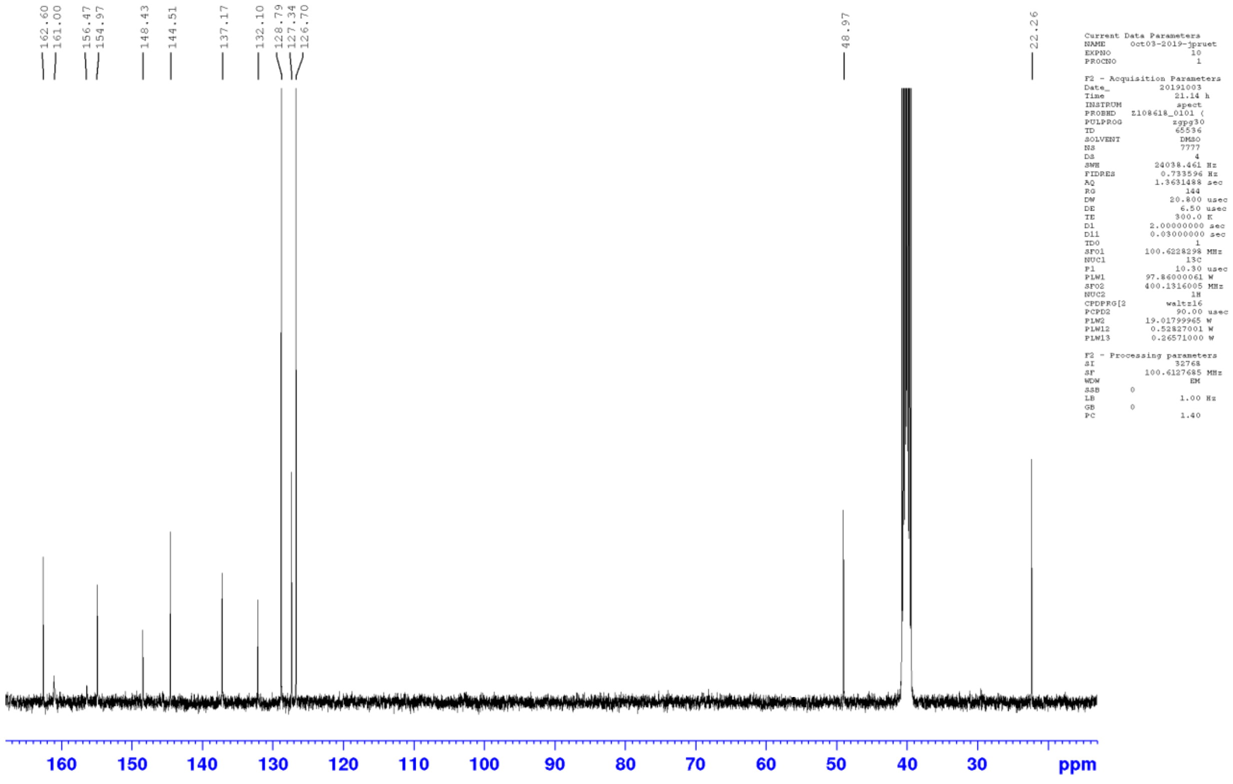

162.60
161.00
156.47
154.97
148.43
144.51
137.17
132.10
128.79
127.34
126.70
48.97
22.26
160 150 140 130 120 110 100 90 80 70 60 50 40 30 ppm
Current Data Parameters
NAME Oct03-2019-jpruet
EXPNO 10
PROCNO 1
F2 - Acquisition Parameters
Date_ 20191003
Time 21.14 h
INSTRUM spect
PROBHD Z108618_0101 (
PULPROG zgpg30
TD 65536
SOLVENT DMSO
NS 7777
DS 4
SWH 24038.461 Hz
FIDRES 0.733596 Hz
AQ 1.3631488 sec
RG 144
DW 20.800 usec
DE 6.50 usec
TE 300.0 K
D1 2.00000000 sec
D11 0.03000000 sec
TD0 1
SFO1 100.6228298 MHz
NUC1 13C
P1 10.30 usec
PLW1 97.86000061 W
SFO2 400.1316005 MHz
NUC2 1H
CPDPRG[2 waltz16
PCPD2 90.00 usec
PLW2 19.01799965 W
PLW12 0.52827001 W
PLW13 0.26571000 W
F2 - Processing parameters
SI 32768
SF 100.6127685 MHz
WDW EM
SSB 0
LB 1.00 Hz
GB 0
PC 1.40

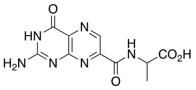

13 (in DMSO-d_6)

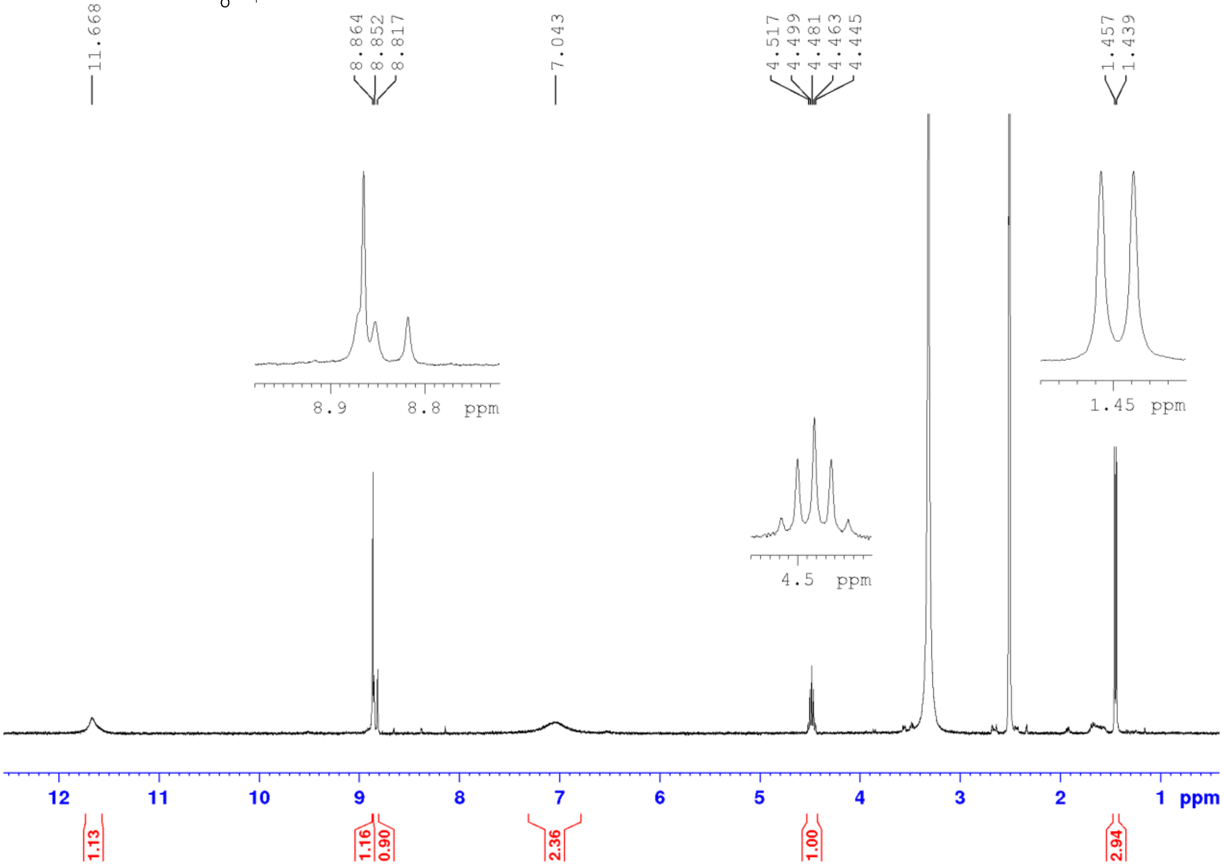

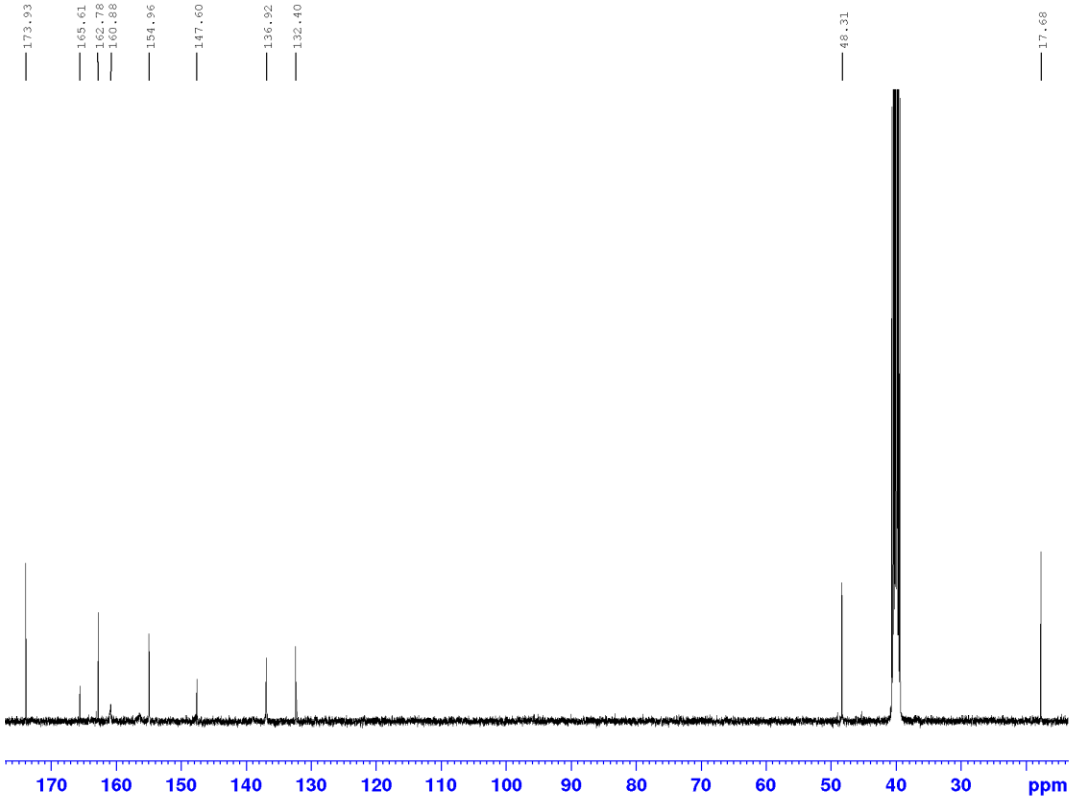


```
Current Data Parameters
NAME      Aug30-2019-jpruet
EXPNO                    15
PROCNO                    1

F2 - Acquisition Parameters
Date_              20190831
Time                  0.09 h
INSTRUM               spect
PROBHD   Z108618_0101 (
PULPROG             zgpg30
TD                   65536
SOLVENT               DMSO
NS                    5888
DS                       4
SWH              24038.461 Hz
FIDRES            0.733596 Hz
AQ               1.3631488 sec
RG                     114
DW                  20.800 usec
DE                    6.50 usec
TE                   300.0 K
D1              2.00000000 sec
D11             0.03000000 sec
TD0                      1
SFO1           100.6228298 MHz
NUC1                   13C
P1                   10.30 usec
PLW1           97.86000061 W
SFO2           400.1316005 MHz
NUC2                    1H
CPDPRG[2           waltz16
PCPD2                90.00 usec
PLW2           19.01799965 W
PLW12           0.52827001 W
PLW13           0.26571000 W

F2 - Processing parameters
SI                   32768
SF             100.6127690 MHz
WDW                     EM
SSB       0
LB                    1.00 Hz
GB        0
PC                    1.40
```

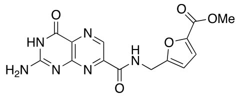
O
HN
N
H2N
N
N
H
N
O
O
O
OMe

14 (in DMSO-d_6)

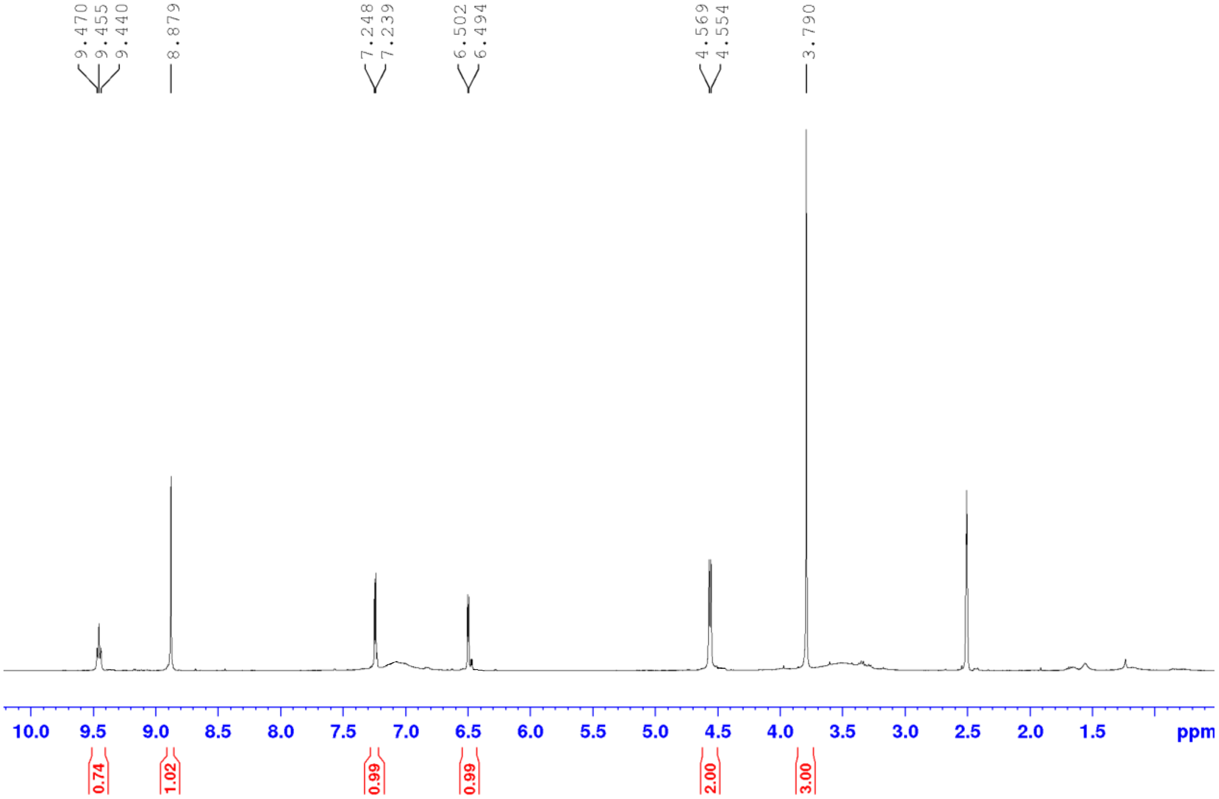
9.470
9.455
9.440
8.879
7.248
7.239
6.502
6.494
4.569
4.554
3.790
10.0
9.5
9.0
8.5
8.0
7.5
7.0
6.5
6.0
5.5
5.0
4.5
4.0
3.5
3.0
2.5
2.0
1.5
ppm
0.74
1.02
0.99
0.99
2.00
3.00

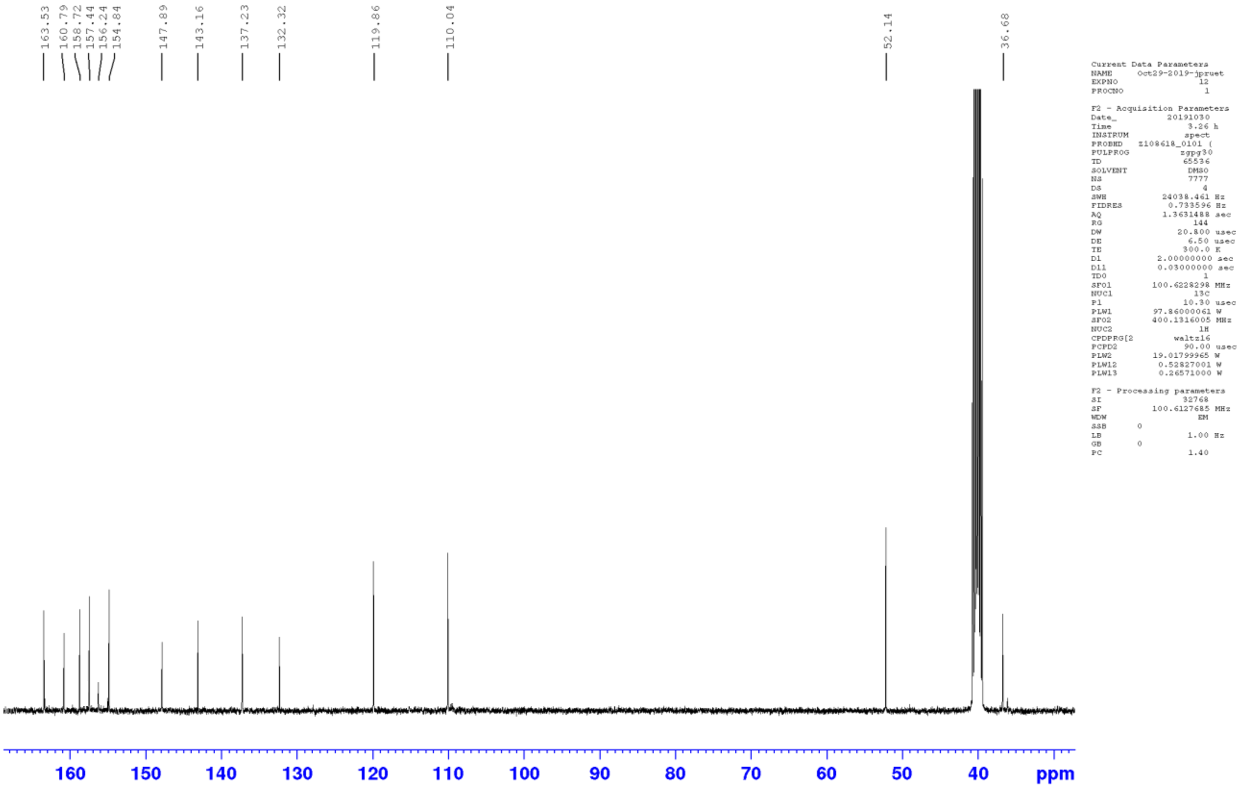
163.53
160.79
158.72
157.44
156.24
154.84
147.89
143.16
137.23
132.32
119.86
110.04
52.14
36.68
160
150
140
130
120
110
100
90
80
70
60
50
40
ppm
Current Data Parameters
NAME Oct29-2019-jpruet
EXPNO 12
PROCNO 1
F2 - Acquisition Parameters
Date_ 20191030
Time 3.26 h
INSTRUM spect
PROBHD Z108618_0101 (
PULPROG zgpg30
TD 65536
SOLVENT DMSO
NS 7777
DS 4
SWH 24038.461 Hz
FIDRES 0.733596 Hz
AQ 1.3631488 sec
RG 144
DW 20.800 usec
DE 6.50 usec
TE 300.0 K
D1 2.00000000 sec
D11 0.03000000 sec
TD0 1
SFO1 100.6228298 MHz
NUC1 13C
P1 10.30 usec
PLW1 97.86000061 W
SFO2 400.1316005 MHz
NUC2 1H
CPDPRG[2 waltz16
PCPD2 90.00 usec
PLW2 19.01799965 W
PLW12 0.52827001 W
PLW13 0.26571000 W
F2 - Processing parameters
SI 32768
SF 100.6127685 MHz
WDW EM
SSB 0
LB 1.00 Hz
GB 0
PC 1.40

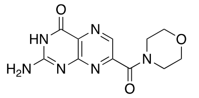
O
HN
N
N
O
H2N
N
N
O

15 (in DMSO-*d*$_6$)

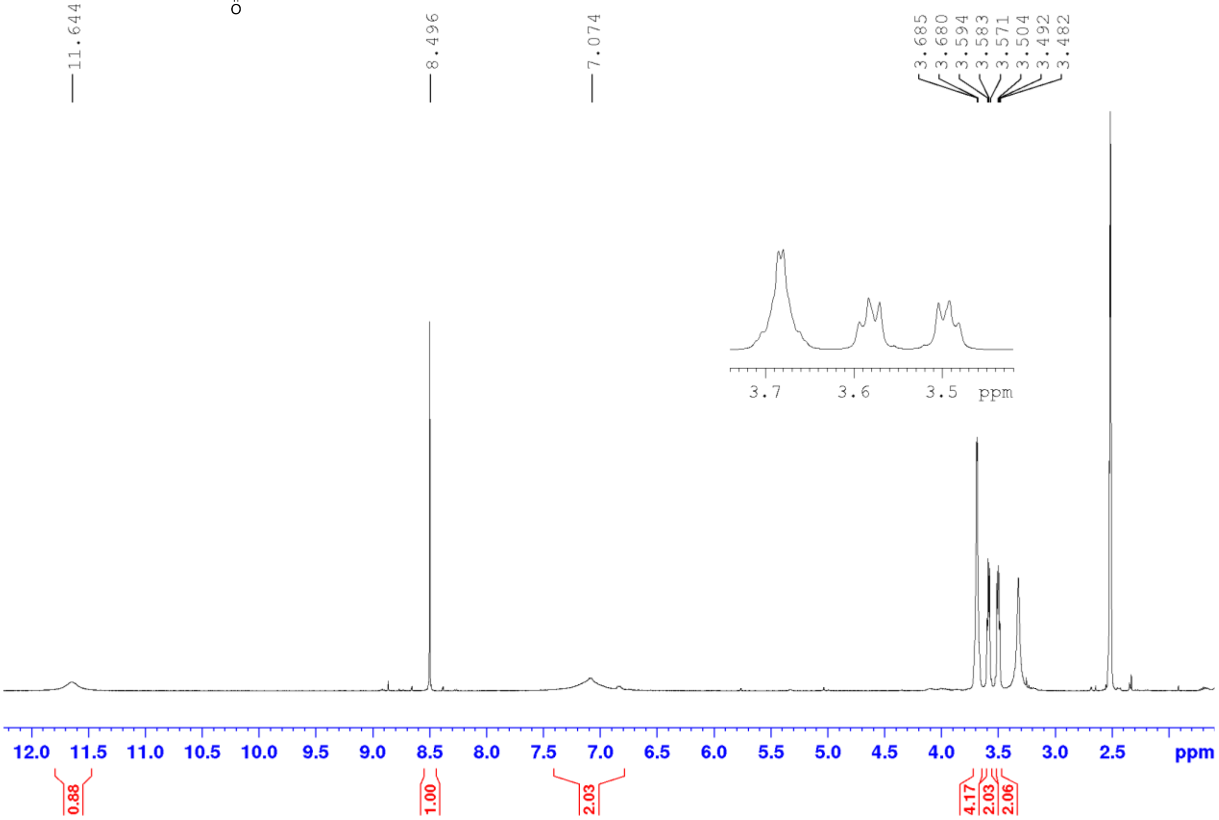
11.644
8.496
7.074
3.685
3.680
3.594
3.583
3.571
3.504
3.492
3.482
3.7
3.6
3.5
ppm
12.0
11.5
11.0
10.5
10.0
9.5
9.0
8.5
8.0
7.5
7.0
6.5
6.0
5.5
5.0
4.5
4.0
3.5
3.0
2.5
ppm
0.88
1.00
2.03
4.17
2.03
2.06

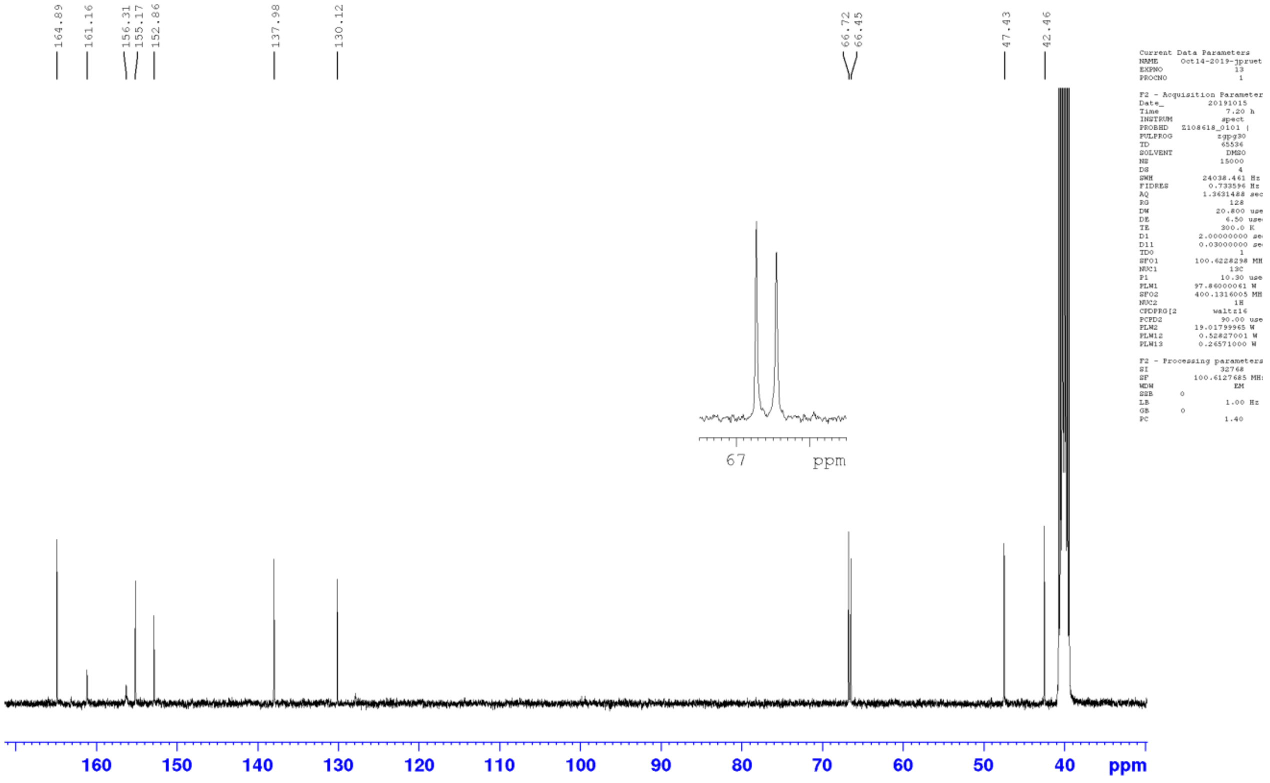
164.89
161.16
156.31
155.17
152.86
137.98
130.12
66.72
66.45
47.43
42.46
67
ppm
160
150
140
130
120
110
100
90
80
70
60
50
40
ppm
Current Data Parameters

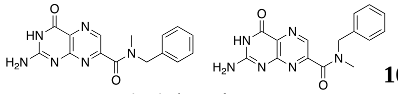

1-to-1 mixture of rotamers

16 (in DMSO-d_6)

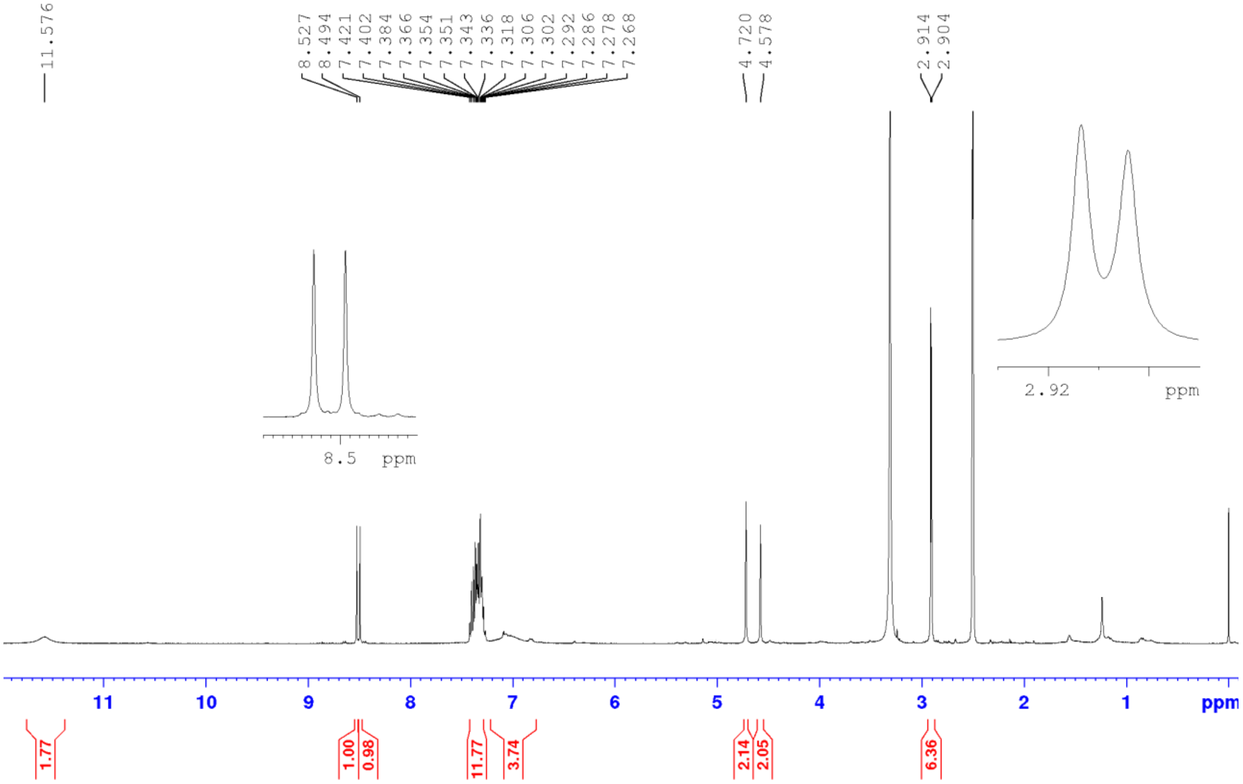

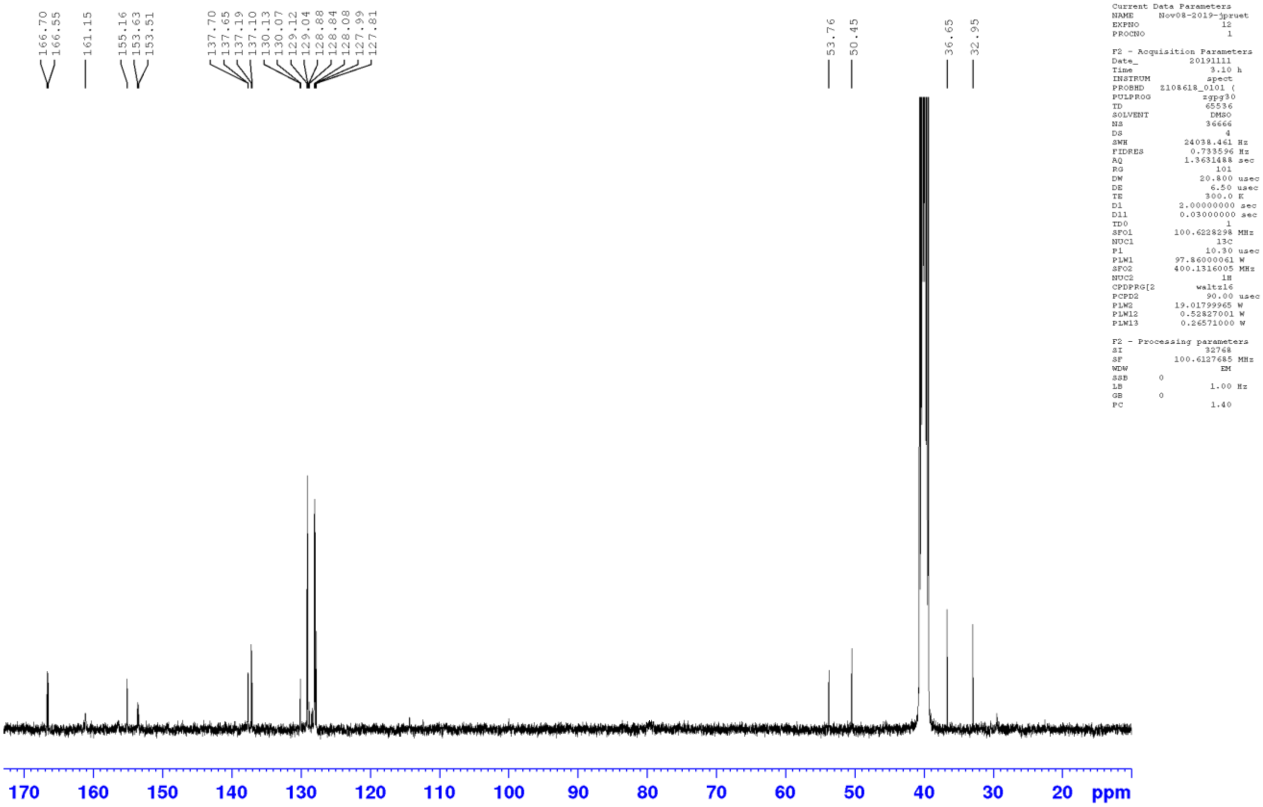

References:

1) Obermayer, D.; Znidar, D.; Glotz, G.; Stadler, A.; Dallinger, D.; Kappe, C. *J. Org. Chem.* 81 **2016** 11788-11801
2) J.M. Pruet, J.D. Robertus, E.V. Anslyn, *Tetrahedron Lett.* 51 **2010** 2539
3) Saito R.; Suzuki S.; Sasaki K. *Bioorg Med Chem Lett* **2016**, *26*: 4870.
4) This way of indicating amide rotamers in NMR was used in the following report: X. Li; S. Danishefsky, *J. Am. Chem. Soc. 130* **2008,** 5446
5) T.K. Chakraborty, S. Tapadar, S.K. Kumar, *Tetrahedron Lett.* 43 **2002**, 1317
6) E.V. Anslyn, D. Dougherty, *Modern Physical Organic Chemistry*; University Science Books, **2006**, p 387
